# Supplementary material for: Interaction of G2019S LRRK2 and metabolic syndrome in a two-hit mouse model of Parkinson’s disease: LRRK2-driven systemic depletion of pyrimidine nucleosides
Source: NPJ Parkinsons Dis. 2026 Jul 8;12:265. doi: 10.1038/s41531-026-01465-x (PMC13724748; doi:10.1038/s41531-026-01465-x)

Supplementary Figures

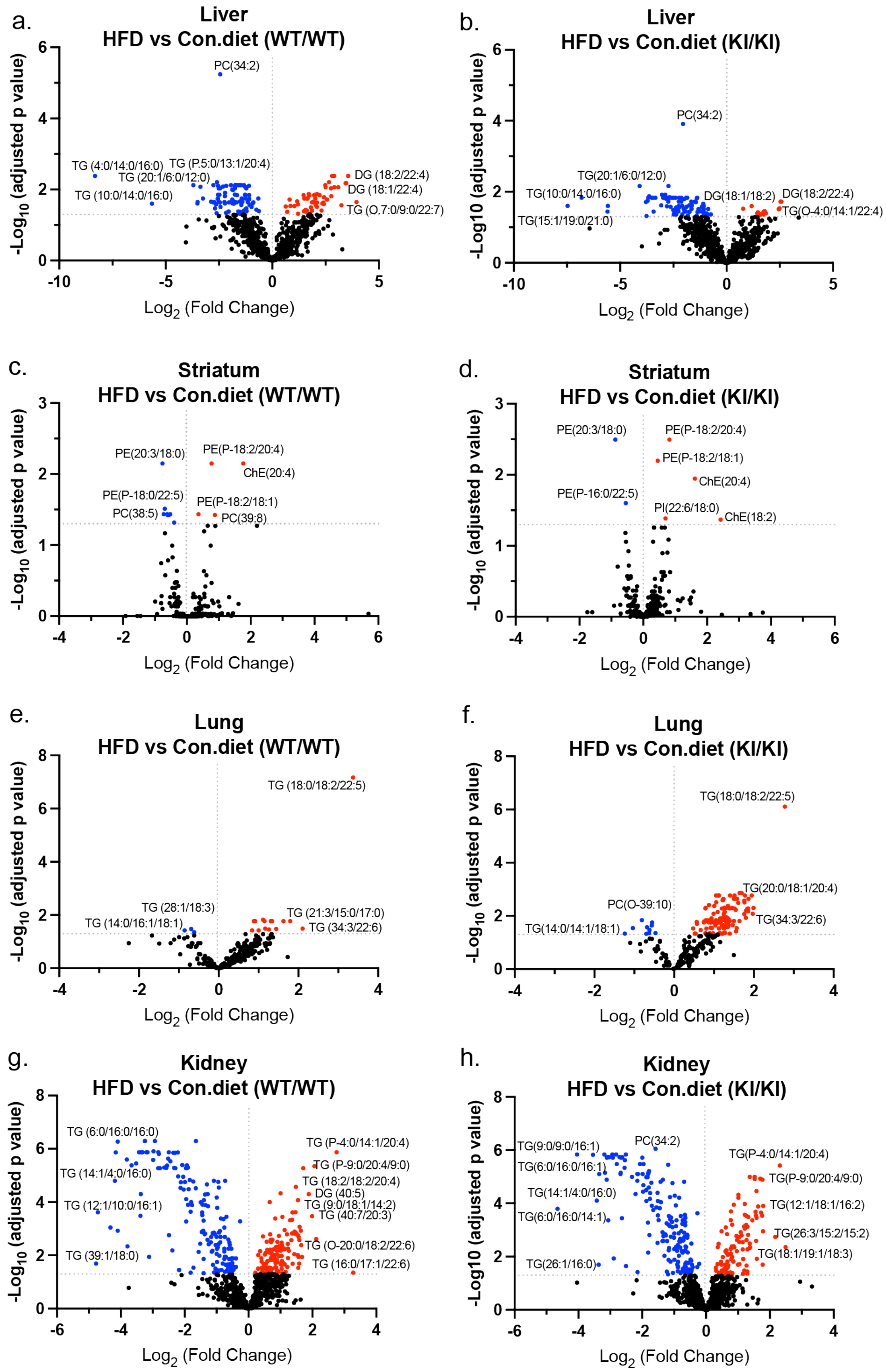

Figure S1. Lipid alterations across tissues in *G2019S LRRK2* knockin and wild-type mice under HFD. Volcano plot showing differences in the abundance of lipids between HFD and Con. diet in liver (**a-b**), striatum (**c-d**), lung (**e-f**), and kidney (**g-h**) of *G2019S LRRK2* knockin mice and wild-type controls. WT/WT, wild-type mice; KI/KI, homozygous *G2019S LRRK2* knockin mice. Each point represents a lipid species. X-

axis shows $\log_2$ (fold change) in lipid abundance between high-fat diet and control diet groups. Y-axis shows $-\log_{10}$ (adjusted *p*-value) from limma's empirical Bayes moderated analysis (eBayes) combined with Benjamini-Hochberg (BH) for multiple testing correction ($n = 6$ per group). Lipids with adjusted $P<0.05$ are considered significantly altered. Significantly upregulated lipids are highlighted in red and significantly downregulated lipids are highlighted in blue.

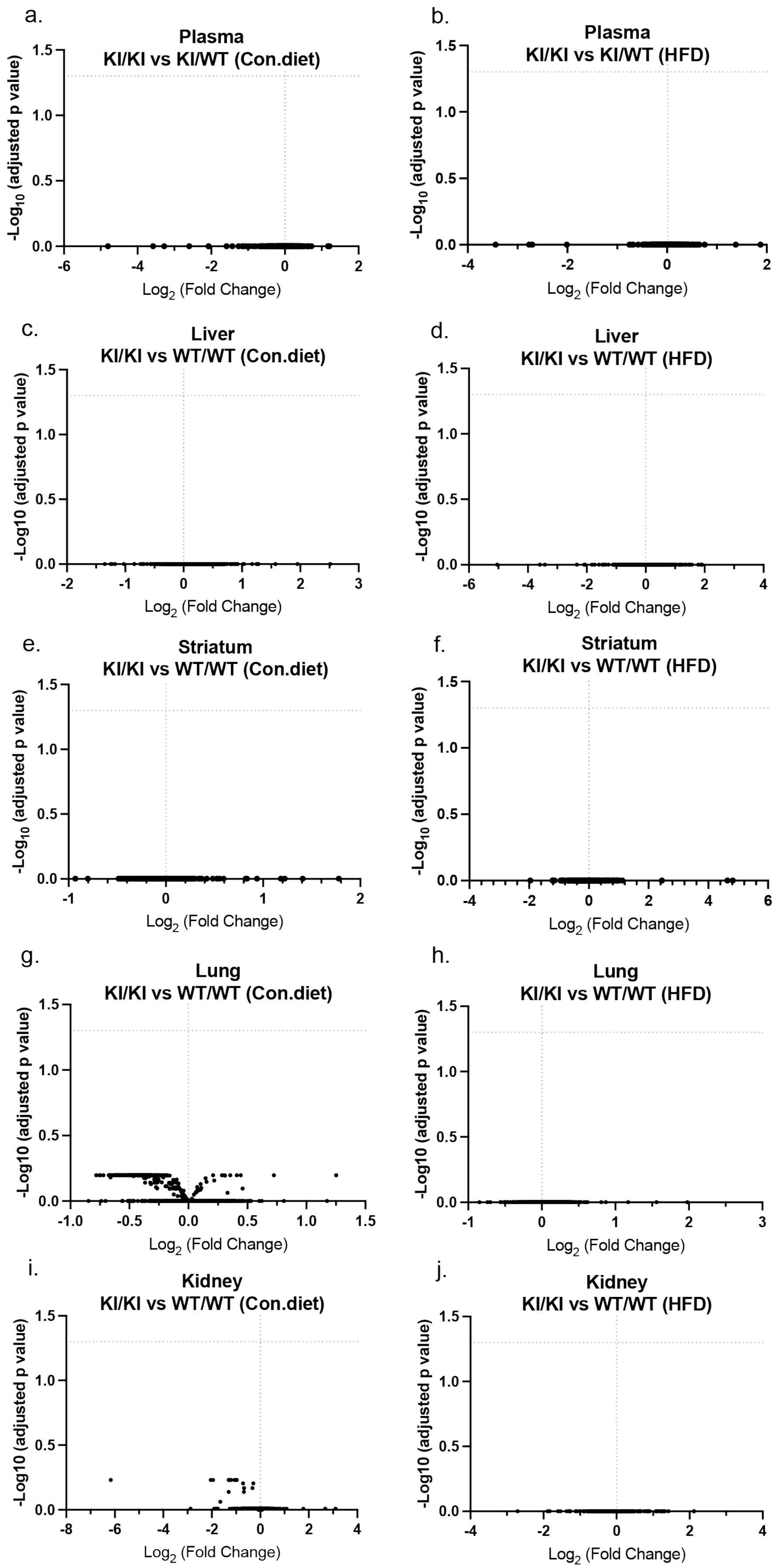
a.
Plasma
KI/KI vs KI/WT (Con.diet)
-Log10 (adjusted p value)
Log2 (Fold Change)
b.
Plasma
KI/KI vs KI/WT (HFD)
-Log10 (adjusted p value)
Log2 (Fold Change)
c.
Liver
KI/KI vs WT/WT (Con.diet)
-Log10 (adjusted p value)
Log2 (Fold Change)
d.
Liver
KI/KI vs WT/WT (HFD)
-Log10 (adjusted p value)
Log2 (Fold Change)
e.
Striatum
KI/KI vs WT/WT (Con.diet)
-Log10 (adjusted p value)
Log2 (Fold Change)
f.
Striatum
KI/KI vs WT/WT (HFD)
-Log10 (adjusted p value)
Log2 (Fold Change)
g.
Lung
KI/KI vs WT/WT (Con.diet)
-Log10 (adjusted p value)
Log2 (Fold Change)
h.
Lung
KI/KI vs WT/WT (HFD)
-Log10 (adjusted p value)
Log2 (Fold Change)
i.
Kidney
KI/KI vs WT/WT (Con.diet)
-Log10 (adjusted p value)
Log2 (Fold Change)
j.
Kidney
KI/KI vs WT/WT (HFD)
-Log10 (adjusted p value)
Log2 (Fold Change)

Figure S2. Lipid alterations across tissues in *G2019S LRRK2* knockin mice under HFD and control diet. Volcano plot shows differences in the abundance of lipids between *G2019S LRRK2* knockin and wild-type mice in the plasma (a, b), liver (c-d), striatum (**e-f**), lung (**g-h)**, and kidney (**i-j**) under HFD and Con. diet. WT/WT, wild-type mice; KI/KI, homozygous *G2019S LRRK2* knockin mice. Each point represents a lipid species. X-axis shows $\log_2$ (fold change) in lipid abundance between high-fat diet and control diet groups. Y-axis shows $-\log_{10}$ (adjusted *p*-value) from limma's empirical Bayes moderated analysis (eBayes) combined with Benjamini-Hochberg (BH) for multiple testing correction (n = 6 per group). Lipids with adjusted P<0.05 are considered significantly altered. No significant alterations were found.

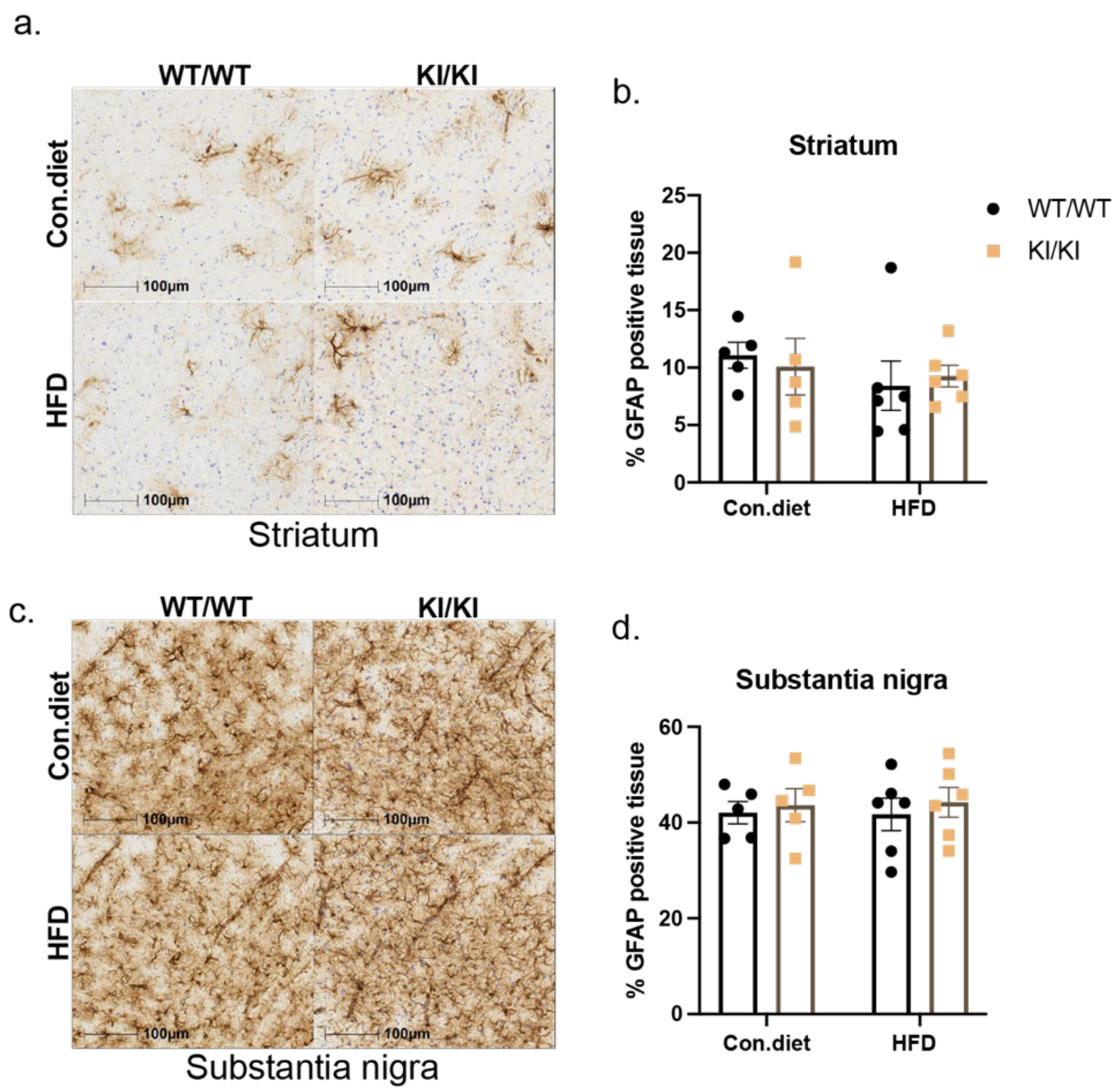

Figure S3. Pre-existing metabolic syndrome fails to induce reactive astrogliosis in striatum and substantia nigra of *G2019S LRRK2* knockin mice. a, c Representative image of GFAP immunostaining in the striatum (a) and substantia nigra (c) showing astrocytes distribution. Scale bar: 100 μm. **b, d** Quantification of GFAP-positive area in the striatum (b) and substantia nigra (d) (*n* = 5-6 per group). WT/WT, wild-type mice; KI/KI, homozygous *G2019S LRRK2* knockin mice. Data are presented as mean ± SEM. A two-way ANOVA was performed to evaluate the effects of high-fat diet and *G2019S LRRK2* and their interaction followed by Fisher's LSD comparisons. No significant effects were found.

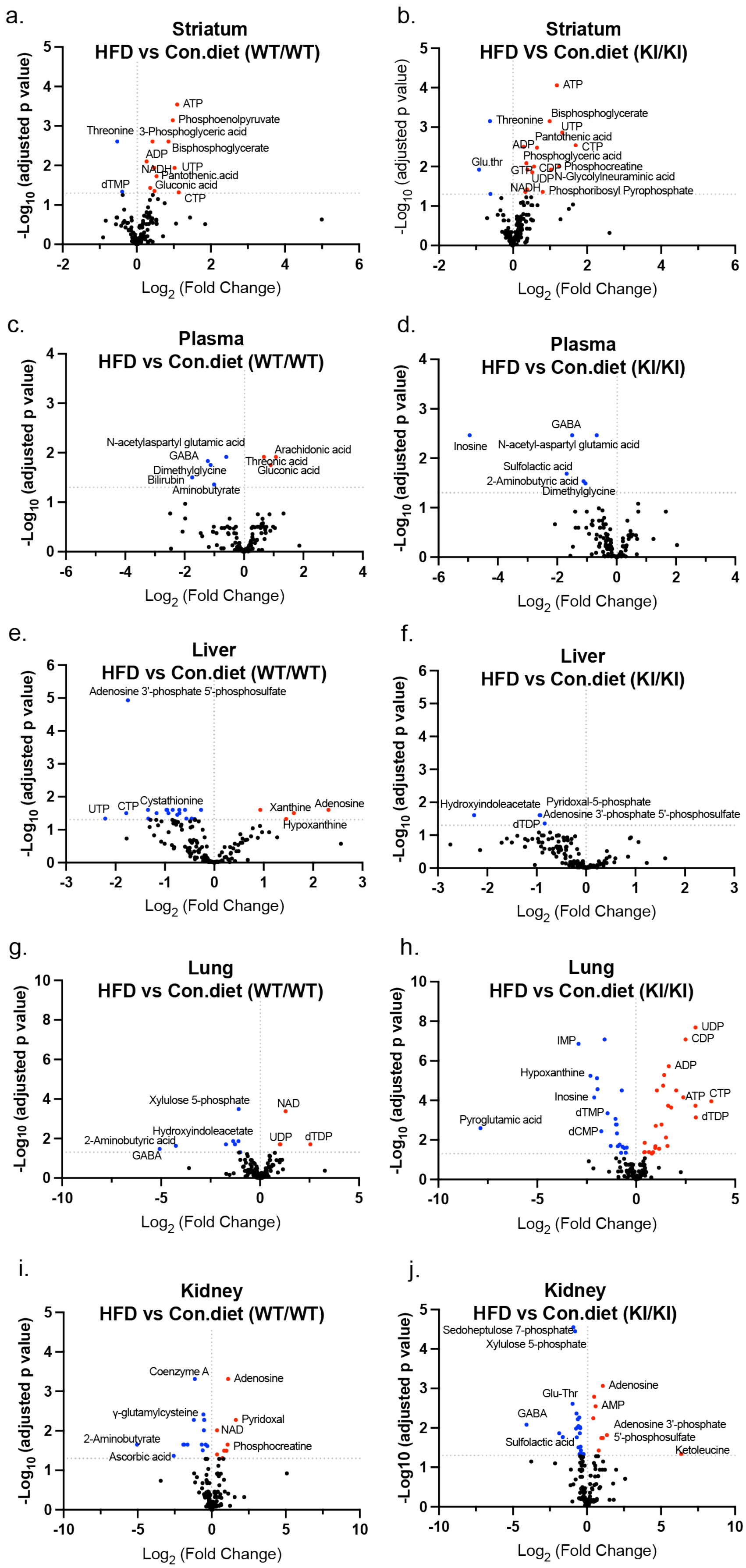
a.
Striatum
HFD vs Con.diet (WT/WT)
-Log10 (adjusted p value)
Log2 (Fold Change)
ATP
Phosphoenolpyruvate
Threonine
3-Phosphoglyceric acid
Bisphosphoglycerate
ADP
NADH
UTP
Pantothenic acid
Gluconic acid
dTMP
CTP
b.
Striatum
HFD VS Con.diet (KI/KI)
-Log10 (adjusted p value)
Log2 (Fold Change)
ATP
Bisphosphoglycerate
Threonine
UTP
Pantothenic acid
ADP
CTP
Phosphoglyceric acid
Glu.thr
GTP
CDP
Phosphocreatine
UDP
N-Glycolylneuraminic acid
NADH
Phosphoribosyl Pyrophosphate
c.
Plasma
HFD vs Con.diet (WT/WT)
-Log10 (adjusted p value)
Log2 (Fold Change)
N-acetylaspartyl glutamic acid
GABA
Arachidonic acid
Threonic acid
Gluconic acid
Dimethylglycine
Bilirubin
Aminobutyrate
d.
Plasma
HFD vs Con.diet (KI/KI)
-Log10 (adjusted p value)
Log2 (Fold Change)
GABA
Inosine
N-acetyl-aspartyl glutamic acid
Sulfolactic acid
2-Aminobutyric acid
Dimethylglycine
e.
Liver
HFD vs Con.diet (WT/WT)
-Log10 (adjusted p value)
Log2 (Fold Change)
Adenosine 3'-phosphate 5'-phosphosulfate
UTP
CTP
Cystathionine
Xanthine
Adenosine
Hypoxanthine
f.
Liver
HFD vs Con.diet (KI/KI)
-Log10 (adjusted p value)
Log2 (Fold Change)
Hydroxyindoleacetate
Pyridoxal-5-phosphate
Adenosine 3'-phosphate 5'-phosphosulfate
dTDP
g.
Lung
HFD vs Con.diet (WT/WT)
-Log10 (adjusted p value)
Log2 (Fold Change)
Xylulose 5-phosphate
NAD
Hydroxyindoleacetate
2-Aminobutyric acid
GABA
UDP
dTDP
h.
Lung
HFD vs Con.diet (KI/KI)
-Log10 (adjusted p value)
Log2 (Fold Change)
UDP
CDP
IMP
ADP
Hypoxanthine
Inosine
ATP
CTP
dTMP
dTDP
Pyroglutamic acid
dCMP
i.
Kidney
HFD vs Con.diet (WT/WT)
-Log10 (adjusted p value)
Log2 (Fold Change)
Coenzyme A
Adenosine
γ-glutamylcysteine
Pyridoxal
NAD
2-Aminobutyrate
Phosphocreatine
Ascorbic acid
j.
Kidney
HFD vs Con.diet (KI/KI)
-Log10 (adjusted p value)
Log2 (Fold Change)
Sedoheptulose 7-phosphate
Xylulose 5-phosphate
Adenosine
Glu-Thr
AMP
GABA
Adenosine 3'-phosphate
5'-phosphosulfate
Sulfolactic acid
Ketoleucine

Figure S4. Non-lipid metabolite alterations across tissues in *G2019S LRRK2* knockin and wild-type mice under HFD. Volcano plot shows differences in the abundance of non-lipid metabolites between HFD and Con. diet groups in *G2019S LRRK2* knockin mice and wild-type mice in the striatum (**a, b**), plasma (**c-d**), liver (**e-f**), lung (**g-h)**, and kidney (**i-j**). WT/WT, wild-type mice; KI/KI, homozygous *G2019S LRRK2* knockin mice. Each point represents a non-lipid metabolite. X-axis shows $\log_2$ (fold change) in non-lipid metabolite abundance between HFD and Con. diet groups. Y-axis shows $-\log_{10}$ (adjusted *p*-value) from limma's empirical Bayes moderated analysis (eBayes) combined with Benjamini-Hochberg (BH) for multiple testing correction (n = 6 per group). Non-lipid metabolites with adjusted $P<0.05$ are considered significantly altered. Significantly upregulated metabolites are highlighted in red and significantly downregulated metabolites are highlighted in blue.

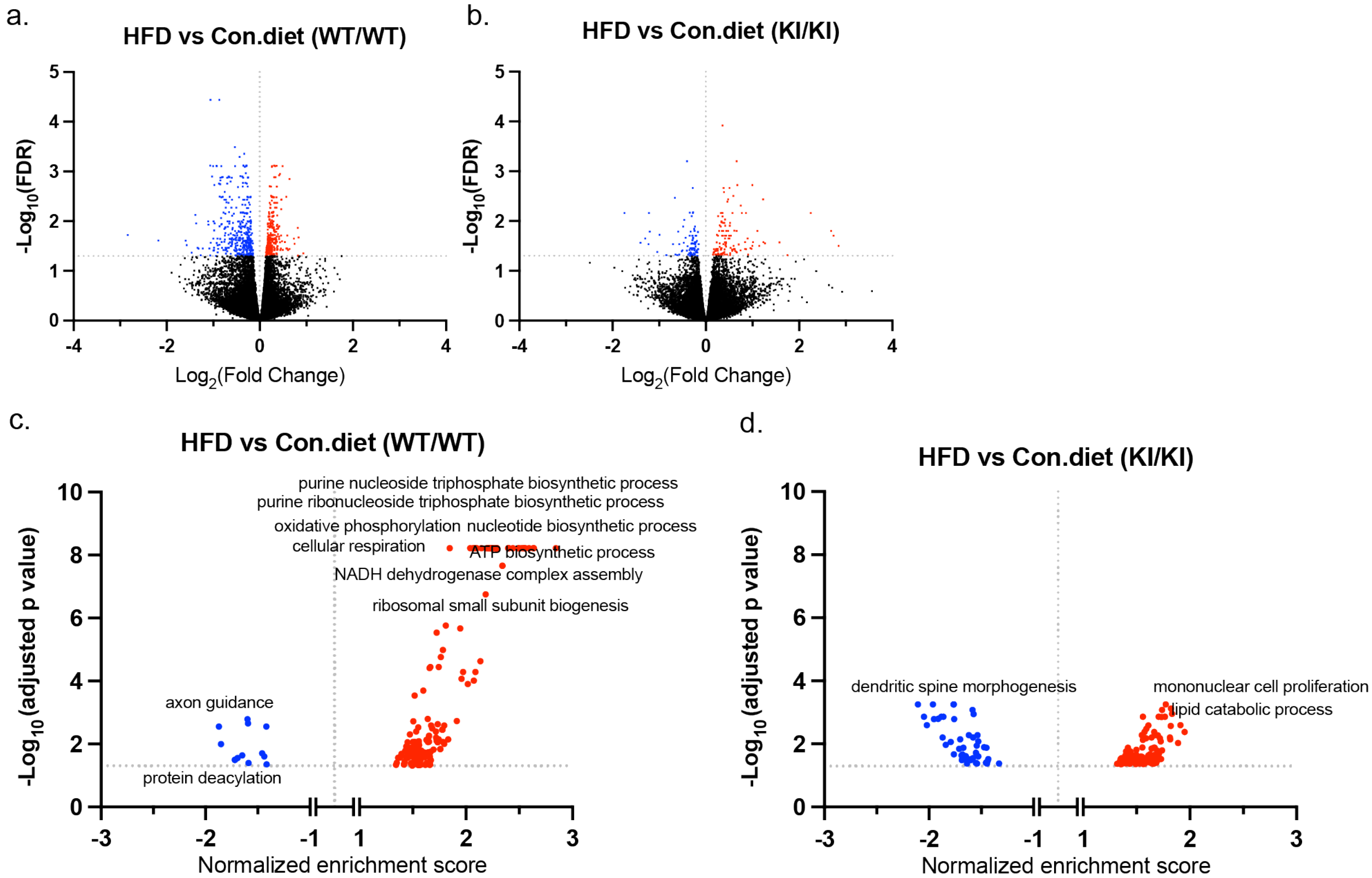

Figure S5. Pre-existing metabolic syndrome reveals altered brain energy metabolism in *G2019S LRRK2* knockin mice. a-b Volcano plot showing differential gene expression in the striatum between HFD and Con.diet in *G2019S LRRK2* knockin and wild-type mice. Each point represents one gene. X-axis shows $\log_2$ (fold change) in gene expression. Y-axis shows $-\log_{10}$ (adjusted *p*-value), significance was defined as a Benjamini–Hochberg adjusted $P<0.05$ (n = 4 per group). Gene expression with adjusted $P<0.05$ are considered significantly altered. Significantly upregulated genes are highlighted in red and significantly downregulated genes are highlighted in blue. **c-d** Volcano plot illustrates enriched biological processes between *G2019S LRRK2* knockin and wild-type mice under Con.diet and HFD conditions as identified by gene set enrichment analysis (GSEA). Each point represents one process, X-axis shows normalized enrichment score, Y-axis shows $-\log_{10}$ (adjusted *p*-value), significance was defined as a Benjamini–Hochberg adjusted $P<0.05$ (n = 4 per group). Significantly upregulated processes are highlighted in red and significantly downregulated processes are highlighted in blue. WT/WT, wild-type mice; KI/KI, homozygous *G2019S LRRK2* knockin mice.

a.

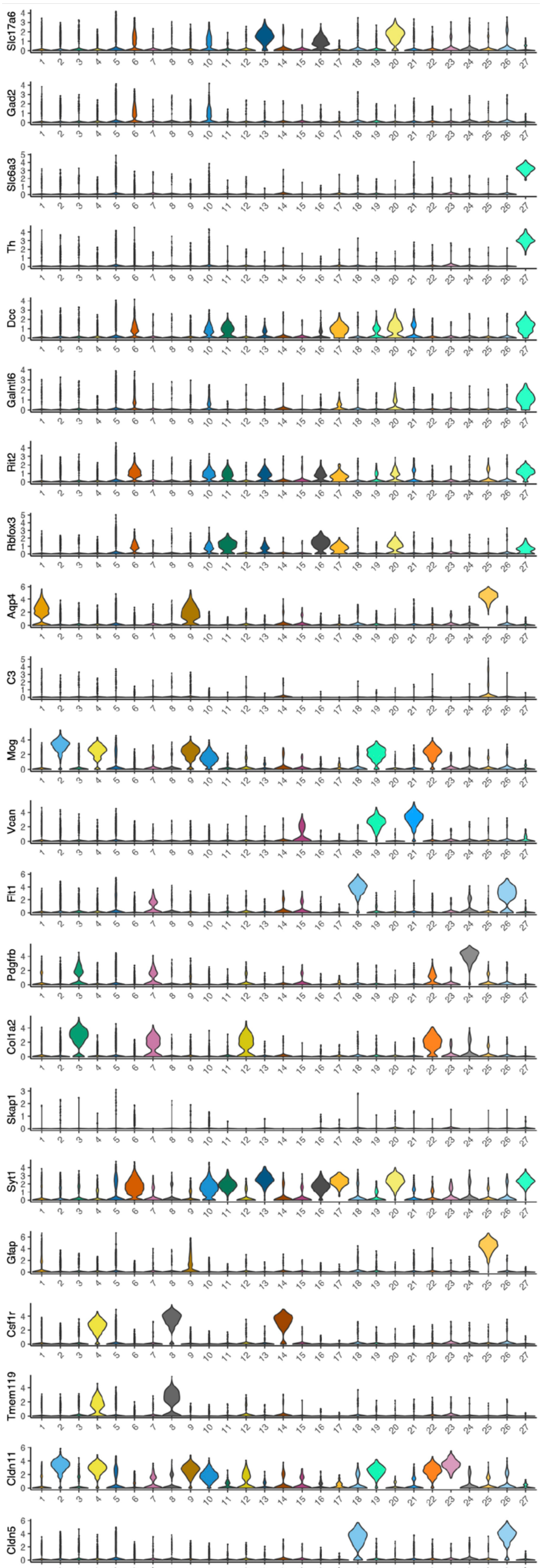

b.

Cell type	Cluster	Counts	Sum
Oligo	2	6301	12293
Oligo	23	96	
Oligo hibrid	22	472	
Oligo hibrid	4	1155	
Oligo hibrid	10	2820	
Oligo hibrid	9	1449	
Neuron	6	6192	8015
Neuron	11	217	
Neuron	13	242	
Neuron	16	577	
Neuron	17	115	
Neuron	20	534	
Neuron	27	138	
Astrocyte	1	3880	4294
Asttrocyte	25	414	
Fibroblast	3	1460	2216
Fibroblast	7	318	
Fibroblast	12	438	
OPC	15	149	1653
OPC	19	387	
OPC	21	1117	
Microglia	8	1115	1297
Microglia	14	182	
Endothelia	18	537	785
Endothelia	26	248	
Pericyte	24	209	209
no annotate	5	3934	3934

Figure S6. Identification of cell type-specific clusters and numbers in the mouse ventral midbrain via snRNA-seq analysis. Nuclei were isolated from ventral midbrain of *G2019S LRRK2* knockin mice and their WT littermates (*n* = 4 mice per group), with 34,969 nuclei recovered in total. **a** Cell type annotation of each cluster using well established cell-type transcript markers. **b** Cell counts and cell type annotated in each cluster.

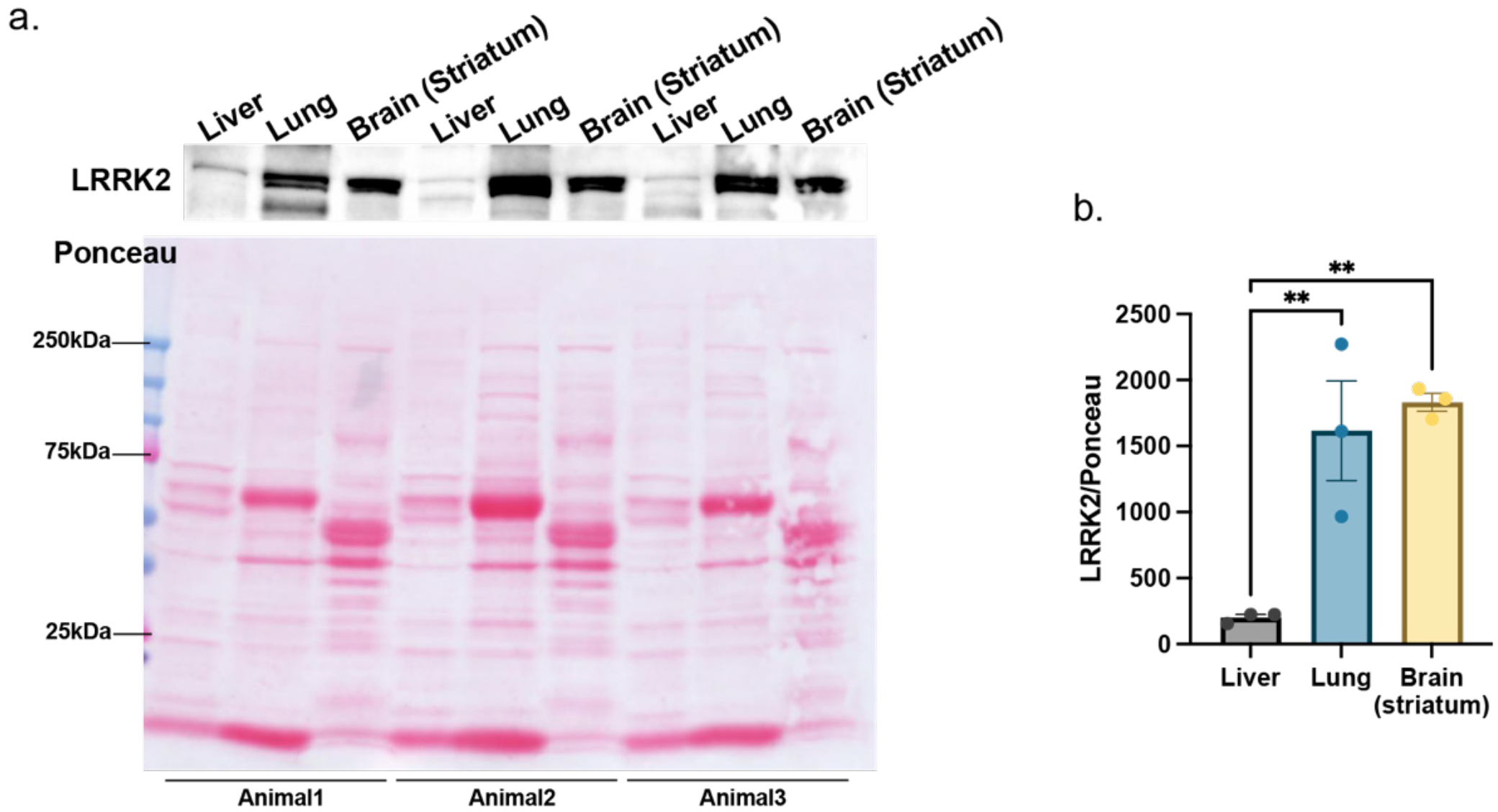

Figure S7. Western blot analysis and quantification of LRRK2 protein levels across tissues. a Sixty µg of total protein from liver, lung, and striatum of adult wild-type mice (*n* = 3 mice/group) were resolved by SDS-PAGE and transferred to nitrocellulose. Membrane was stained with Ponceau S to assess total protein loading, followed by immunoblotting with an anti-LRRK2 antibody. **b** Quantification of LRRK2 protein levels normalized to Ponceau signal. Data are presented as mean ± SEM (*n* = 3 mice/group). One-way ANOVA was performed to evaluate the difference among groups. Significant differences were observed (***P*<0.01) relative to liver.

Supplementary Data

Data S1. Summary of metabolites and lipids identified by metabolomic and lipidomic analysis.

Data S2. Differentially-expressed genes identified by bulk RNA-seq analysis of the striatum.

Data S3. Enriched GO Biological Process (GOBP) terms identified from bulk RNA-seq analysis of the striatum.

Data S4. Differentially-expressed genes identified by single-nucleus RNA-seq analysis of the ventral midbrain.

Data S5. Enriched GO Biological Process (GOBP) terms identified from single-nucleus RNA-seq analysis of the ventral midbrain.

Data S6. Summary of relative protein abundance quantified by mass spectrometry in lung tissue.

Table S1. Antibody information for Western blot (WB) and immunohistochemistry (IHC).

Table S2. Panel of compounds targeted for MS2 fragmentation.

Key Resource Table (KTR).

Table S1. Antibody information for Western blot (WB) and immunohistochemistry (IHC).

**Target (WB)**	**Catalog no.**	**Host**	**Dilution**
LRRK2 (N241A/34)	75-253, NeuroMab	mouse	1:1000
pSer935-LRRK2	ab133450, Abcam	rabbit	1:1000
pSer1292-LRRK2	ab203181, Abcam	rabbit	1:1000
Rab8a	D22D8, Cell Sigal Tech	rabbit	1:1000
pThr72-Rab8a	ab230260, Abcam	rabbit	1:500
Rab10	8127S, Cell Signal Tech	rabbit	1:1000
pThr73-Rab10	ab230261, Abcam	rabbit	1:500
Rab12	18843-1-AP, Proteintech	rabbit	1:1000
pSer106-Rab12	ab256487, Abcam	rabbit	1:1000
Thymidine Phosphorylase	AF7568, R&D systems	sheep	1:200
Actin	MAB1501, Millipore	mouse	1:2000
**Target (IHC)**	**Catalog no.**	**Host**	**Dilution**
Tyrosine Hydroxylase (TH)	N300-109, Novus Biologicals	rabbit	1:2000
Iba1	019-19741, FUJIFILM Wako	rabbit	1:1000
GFAP	G3893, Millipore Sigma	mouse	1:1000
CD68	MCA1957GA, Biorad	rat	1:500
CD3	ab11089, Abcam	rat	1:200

Table S2. Panel of compounds targeted for MS2 fragmentation.

Compound	Formula	Adduct	m/z	z	RT Time (min)	Window (min)
**2,3-Dihydroxy-2-methylbutanoic acid**	C5H10O4	-H	133.0506	1	8.78	4
**D-2-Aminobutyrate**	C4H9NO2	-H	102.0561	1	1.2	2
**Deoxyuridine**	C9H12N2O5	-H	227.0673	1	2.1	4
**2-Hydroxybutyrate**	C4H8O3	-H	103.0401	1	9.36	4
**3-D-Hydroxybutyrate**	C4H8O3	-H	103.0401	1	7.57	4
**3-Hydroxyisobutyrate**	C4H8O3	-H	103.0401	1	7.36	4
**3-Methyl-2-oxovaleric acid**	C6H10O3	-H	129.0557	1	15.58	4
**3-Phosphoglyceric acid**	C3H7O7P	-H	184.9857	1	13.75	4
**4-Hydroxybutyrate**	C4H8O3	-H	103.0401	1	8.65	4
**4-Hydroxyproline**	C5H9NO3	-H	130.051	1	1.16	2
**5-Aminolevulinic acid**	C5H9NO3	-H	130.051	1	1.15	2
**Phosphoribosyl pyrophosphate**	C5H13O14P3	-H	388.9445	1	16.11	4
**Adenine**	C5H5N5	-H	134.0472	1	2.5	3
**Adenosine**	C10H13N5O4	-H	266.0895	1	5.6	4
**Adenosine 3-phosphate 5-phosphosulfate**	C10H15N5O13P2S	-H	505.979	1	16.26	4
**ADP-Ribose**	C15H23N5O14P2	-H	558.0644	1	13.94	4
**cAMP**	C10H12N5O6P	-H	328.0452	1	12.49	4
**ADP**	C10H15N5O10P2	-H	426.0221	1	14.34	4
**AICAR**	C9H15N4O8P	-H	337.0555	1	9.86	4
**Alanine**	C3H7NO2	-H	88.0404	1	1.18	2
**alpha-Ketoglutarate**	C5H6O5	-H	145.0142	1	13.31	4
**AMP**	C10H14N5O7P	-H	346.0558	1	11.11	4
**Arachidonic acid**	C20H32O2	-H	303.233	1	20.054	4
**Ascorbic acid**	C6H8O6	-H	175.0248	1	14.16	4
**Asparagine**	C4H8N2O3	-H	131.0462	1	1.15	2
**Aspartate**	C4H7NO4	-H	132.0302	1	3.99	4
**Asp-Asp**	C8H12N2O7	-H	247.0572	1	12.388	4
**Asp-Glu**	C9H14N2O7	-H	261.0728	1	12.3	4
**Asp-Gly**	C6H10N2O5	-H	189.0517	1	3.741	4
**ATP**	C10H16N5O13P3	-H	505.9885	1	16.2	4
**Bilirubin**	C33H36N4O6	-H	583.2562	1	21.71	4
**Bisphosphoglycerate**	C3H8O10P2	-H	264.952	1	16.18	4

**CDP**	C9H15N3O11P2	-H	402.0109	1	13.48	4
**CDP-ethanolamine**	C11H20N4O11P2	-H	445.0531	1	5.4	4
**CDP-n-acetylneuraminic acid**	C20H31N4O16P	-H	613.14	1	13.309	4
**Citraconic acid**	C5H6O4	-H	129.0193	1	13.3	4
**Citrate**	C6H8O7	-H	191.0197	1	13.92	4
**Citrulline**	C6H13N3O3	-H	174.0884	1	1.18	2
**CMP**	C9H14N3O8P	-H	322.0446	1	8.64	4
**Coenzyme A**	C21H36N7O16P3S	-H	766.1079	1	16.9	4
**Creatine**	C4H9N3O2	-H	130.0622	1	1.2	2
**Creatinine**	C4H7N3O	-H	112.0516	1	1.29	2
**CTP**	C9H16N3O14P3	-H	481.9772	1	15.92	4
**Cystathionine**	C7H14N2O4S	-H	221.0602	1	1.15	2
**Cytidine**	C9H13N3O5	-H	242.0782	1	1.62	2
**Cytosine**	C4H5N3O	-H	110.036	1	1.15	2
**D-2-Hydroxyglutaric acid**	C5H8O5	-H	147.0299	1	12.85	4
**dCMP**	C9H14N3O7P	-H	306.0497	1	9.635	4
**Dihydroorotic acid**	C5H6N2O4	-H	157.0255	1	6.33	4
**Dihydroxyacetone phosphate**	C3H7O6P	-H	168.9907	1	9.39	4
**Dimethylglycine**	C4H9NO2	-H	102.0561	1	1.15	2
**Dopamine**	C8H11NO2	-H	152.0717	1	1.08	2
**dTDP**	C10H16N2O11P2	-H	401.0157	1	14.38	4
**dTMP**	C10H15N2O8P	-H	321.0493	1	10.91	4
**dTTP**	C10H17N2O14P3	-H	480.982	1	16.26	4
**FA 16:0**	C16H32O2	-H	255.233	1	22.34	3
**FA 16:1**	C16H30O2	-H	253.2173	1	21.91	4
**FA 18:0**	C18H36O2	-H	283.2643	1	23.07	1.5
**FA 18:1**	C18H34O2	-H	281.2486	1	22.44	2
**FA 18:2**	C18H32O2	-H	279.233	1	22.05	2
**FA 18:3**	C18H30O2	-H	277.2173	1	21.77	4
**FA 20:1**	C20H38O2	-H	309.2799	1	23.14	1
**FA 20:2**	C20H36O2	-H	307.2643	1	22.65	2
**FA 20:3**	C20H34O2	-H	305.2486	1	22.23	3
**FA 22:2**	C22H40O2	-H	335.2956	1	23.34	1
**FA 22:5**	C22H34O2	-H	329.2486	1	21.99	4
**FA 22:6**	C22H32O2	-H	327.233	1	21.91	4
**Farnesyl pyrophosphate**	C15H28O7P2	-H	381.1237	1	20.64	4
**FAD**	C27H33N9O15P2	-H	784.1499	1	16.05	4
**FMN**	C17H21N4O9P	-H	455.0973	1	14.81	4

**Folic acid**	C19H19N7O6	-H	440.1324	1	14.23	4
**Fructose 1,6-BP**	C6H14O12P2	-H	338.9888	1	14.004	4
**Fumaric acid**	C4H4O4	-H	115.0037	1	13.7	4
**GABA**	C4H9NO2	-H	102.0561	1	1.17	2
**Galactonic acid**	C6H12O7	-H	195.051	1	4.58	4
**gamma-Glu-Cys**	C8H14N2O5S	-H	249.0551	1	7.486	4
**GDP**	C10H15N5O11P2	-H	442.0171	1	13.83	4
**GDP-Mannose**	C16H25N5O16P2	-H	604.0699	1	13.25	4
**Gln-Glu**	C10H17N3O6	-H	274.1045	1	5.62	4
**Glu-Ala**	C8H14N2O5	-H	217.083	1	7.534	4
**Gluconic acid**	C6H12O7	-H	195.051	1	4.84	4
**Glu-Gly**	C7H12N2O5	-H	203.0673	1	4.672	4
**Glu-Ile**	C11H20N2O5	-H	259.1299	1	12.365	4
**Glutaconic acid**	C5H6O4	-H	129.0193	1	13.69	4
**Glutamate**	C5H9NO4	-H	146.0459	1	3.9	4
**Glutamine**	C5H10N2O3	-H	145.0619	1	1.15	2
**5-L-Glutamyl-taurine**	C7H14N2O6S	-H	253.05	1	5.291	4
**gamma-Glu-Thr**	C9H16N2O6	-H	247.0936	1	5.672	4
**gamma-Glu-Val**	C10H18N2O5	-H	245.1143	1	9.995	4
**Glycerol 3-phosphate**	C3H9O6P	-H	171.0064	1	7.4	4
**Glycine**	C2H5NO2	-H	74.0248	1	1.13	2
**GMP**	C10H14N5O8P	-H	362.0507	1	10.03	4
**GTP**	C10H16N5O14P3	-H	521.9834	1	16.01	4
**Hexose phosphate I**	C6H13O9P	-H	259.0224	1	6.39	4
**Hexose phosphate II**	C6H13O9P	-H	259.0224	1	6.6	4
**Hexose phosphate III**	C6H13O9P	-H	259.0224	1	6.86	4
**Hexose phosphate IV**	C6H13O9P	-H	259.0224	1	7.06	4
**Hexose phosphate IX**	C6H13O9P	-H	259.0224	1	9.4	4
**Hexose phosphate V**	C6H13O9P	-H	259.0224	1	7.19	4
**Hexose phosphate VI**	C6H13O9P	-H	259.0224	1	7.41	4
**Hexose phosphate VII**	C6H13O9P	-H	259.0224	1	8.6	4
**Hexose phosphate VIII**	C6H13O9P	-H	259.0224	1	9.16	4
**Hexose phosphate X**	C6H13O9P	-H	259.0224	1	10.49	4
**Histidine**	C6H9N3O2	-H	154.0622	1	1.04	2
**Hydroxydecanoic acid**	C10H20O3	-H	187.134	1	17.32	4
**Hydroxyhexadecanoic acid**	C16H32O3	-H	271.2279	1	21.58	4
**5-Hydroxyindoleacetic acid**	C10H9NO3	-H	190.051	1	12.09	4
**2-Hydroxystearic acid**	C18H36O3	-H	299.2592	1	22.037	3
**Hypoxanthine**	C5H4N4O	-H	135.0312	1	1.7	2
**IMP**	C10H13N4O8P	-H	347.0398	1	10.01	4

**Indolelactic acid**	C11H11NO3	-H	204.0666	1	15.53	4
**Indoxyl sulfate**	C8H7NO4S	-H	212.0023	1	15.5	4
**Inosine**	C10H12N4O5	-H	267.0735	1	2.5	4
**Isoleucine**	C6H13NO2	-H	130.0874	1	1.87	3
**Itaconate**	C5H6O4	-H	129.0193	1	12.6	4
**Ketoisovalerate**	C5H8O3	-H	115.0401	1	13.84	4
**Ketoleucine**	C6H10O3	-H	129.0557	1	15.92	4
**Lactate**	C3H6O3	-H	89.0244	1	6.918	4
**Leucine**	C6H13NO2	-H	130.0874	1	2	3
**Glutathione (reduced)**	C10H17N3O6S	-H	306.0765	1	7.61	4
**Glutathione (oxidized)**	C20H32N6O12S2	-H	611.1447	1	12.57	4
**Malate**	C4H6O5	-H	133.0142	1	12.72	4
**Malonic acid**	C3H4O4	-H	103.0037	1	11.72	4
**Methionine**	C5H11NO2S	-H	148.0438	1	1.6	2
**2-Methylcitrate**	C7H10O7	-H	205.0354	1	14.38	4
**5'-Methylthioadenosine**	C11H15N5O3S	-H	296.0823	1	10.88	4
**N-Acetyl-D-galactosamine 1-phosphate**	C8H16NO9P	-H	300.049	1	7.92	4
**N-Acetyl-1-aspartylglutamic acid**	C11H16N2O8	-H	303.0834	1	15.255	4
**N-Acetyl-L-alanine**	C5H9NO3	-H	130.051	1	8.17	4
**N-Acetyl-D-Glucosamine 6-Phosphate**	C8H16NO9P	-H	300.049	1	8.198	4
**N-Acetyl-L-glutamic acid**	C7H11NO5	-H	188.0564	1	13.15	4
**N-Acetyl-L-aspartic acid**	C6H9NO5	-H	174.0408	1	13.05	4
**N-Acetylglutamine**	C7H12N2O4	-H	187.0724	1	6.859	4
**N-Acetyl-L-methionine**	C7H13NO3S	-H	190.0543	1	13.1	4
**N-Acetylneuraminic acid**	C11H19NO9	-H	308.0987	1	5.717	4
**N-Acetyl-L-phenylalanine**	C11H13NO3	-H	206.0823	1	15.61	4
**N-Acetylserine**	C5H9NO4	-H	146.0459	1	5.9	4
**N-Acetyltryptophan**	C13H14N2O3	-H	245.0932	1	15.56	4
**NAD+**	C21H27N7O14P2	-H	662.1018	1	8.61	4
**NADH**	C21H29N7O14P2	-H	664.1175	1	14.41	4
**NADP+**	C21H28N7O17P3	-H	742.0682	1	13.81	4
**NADPH**	C21H30N7O17P3	-H	744.0838	1	16.18	4
**Carglumic acid**	C6H10N2O5	-H	189.0517	1	12.61	4

**N-Glycolylneuraminic acid**	C11H19NO10	-H	324.0936	1	4.95	4
**O-Acetylserine**	C5H9NO4	-H	146.0459	1	6.37	4
**O-Phosphoethanolamine**	C2H8NO4P	-H	140.0118	1	1.65	3
**Ophthalmate**	C11H19N3O6	-H	288.1201	1	7.85	4
**Orotic acid**	C5H4N2O4	-H	155.0098	1	8.15	4
**Orotidine**	C10H12N2O8	-H	287.0521	1	7.221	4
**Pantothenic acid**	C9H17NO5	-H	218.1034	1	11.17	4
**Pentose phosphate I**	C5H11O8P	-H	229.0119	1	6.82	4
**Pentose phosphate III**	C5H11O8P	-H	229.0119	1	8	4
**Pentose phosphate IV**	C5H11O8P	-H	229.0119	1	8.7	4
**Pentose phosphate V**	C5H11O8P	-H	229.0119	1	10.33	4
**Pentose phosphate II**	C5H11O8P	-H	229.0119	1	7.8	4
**Phenylalanine**	C9H11NO2	-H	164.0717	1	3.75	4
**Phenyllactic acid**	C9H10O3	-H	165.0557	1	15.61	4
**Phosphocreatine**	C4H10N3O5P	-H	210.0285	1	12.52	4
**Phosphoenolpyruvate**	C3H5O6P	-H	166.9751	1	14.29	4
**Phosphoserine**	C3H8NO6P	-H	184.0016	1	8.79	4
**Hydroxyphenyllactic acid**	C9H10O4	-H	181.0506	1	12.06	4
**Proline**	C5H9NO2	-H	114.0561	1	1.22	2
**Pyridoxal**	C8H9NO3	-H	166.051	1	2.06	3
**Pyridoxal 5'-phosphate**	C8H10NO6P	-H	246.0173	1	13.95	4
**Pyridoxine**	C8H11NO3	-H	168.0666	1	2.13	3
**Pyroglutamic acid**	C5H7NO3	-H	128.0353	1	7.065	4
**Pyruvate**	C3H4O3	-H	87.0088	1	8.64	4
**Riboflavin**	C17H20N4O6	-H	375.131	1	11.9	4
**SAH**	C14H20N6O5S	-H	383.1143	1	4.55	4
**Sedoheptulose 7-phosphate**	C7H15O10P	-H	289.033	1	7.045	4
**Serine**	C3H7NO3	-H	104.0353	1	1.14	2
**S-Lactoylglutathione**	C13H21N3O8S	-H	378.0977	1	13.616	4
**Succinate**	C4H6O4	-H	117.0193	1	12.05	4
**Taurine**	C2H7NO3S	-H	124.0074	1	1.18	2
**Threonic acid**	C4H8O5	-H	135.0299	1	5.01	4
**Threonine**	C4H9NO3	-H	118.051	1	1.17	2
**Thymidine**	C10H14N2O5	-H	241.083	1	4.4	4
**Tryptophan**	C11H12N2O2	-H	203.0826	1	7.422	4
**Tyrosine**	C9H11NO3	-H	180.0666	1	1.95	3
**UDP**	C9H14N2O12P2	-H	402.9949	1	13.82	4

**UDP-alpha-D-glucuronic acid**	C15H22N2O18P2	-H	579.027	1	15.88	4
**UDP-hexose**	C15H25N2O17P2	-H	566.0556	1	13.29	4
**UDP-N-acetylhexosamine**	C17H27N3O17P2	-H	606.0743	1	13.37	4
**UMP**	C9H13N2O9P	-H	323.0286	1	9.65	4
**Uracil**	C4H4N2O2	-H	111.02	1	1.49	2
**Ureidosuccinic acid**	C5H8N2O5	-H	175.036	1	12.58	4
**Uric acid**	C5H4N4O3	-H	167.0211	1	5.07	4
**Uridine**	C9H12N2O6	-H	243.0623	1	1.95	3
**UTP**	C9H15N2O15P3	-H	482.9613	1	16.09	4
**Valine**	C5H11NO2	-H	116.0717	1	1.35	2
**Xanthine**	C5H4N4O2	-H	151.0261	1	1.7	2
**Xanthosine**	C10H12N4O6	-H	283.0684	1	7.28	4

Uncropped Western blots

From Fig. 4g

Total LRRK2

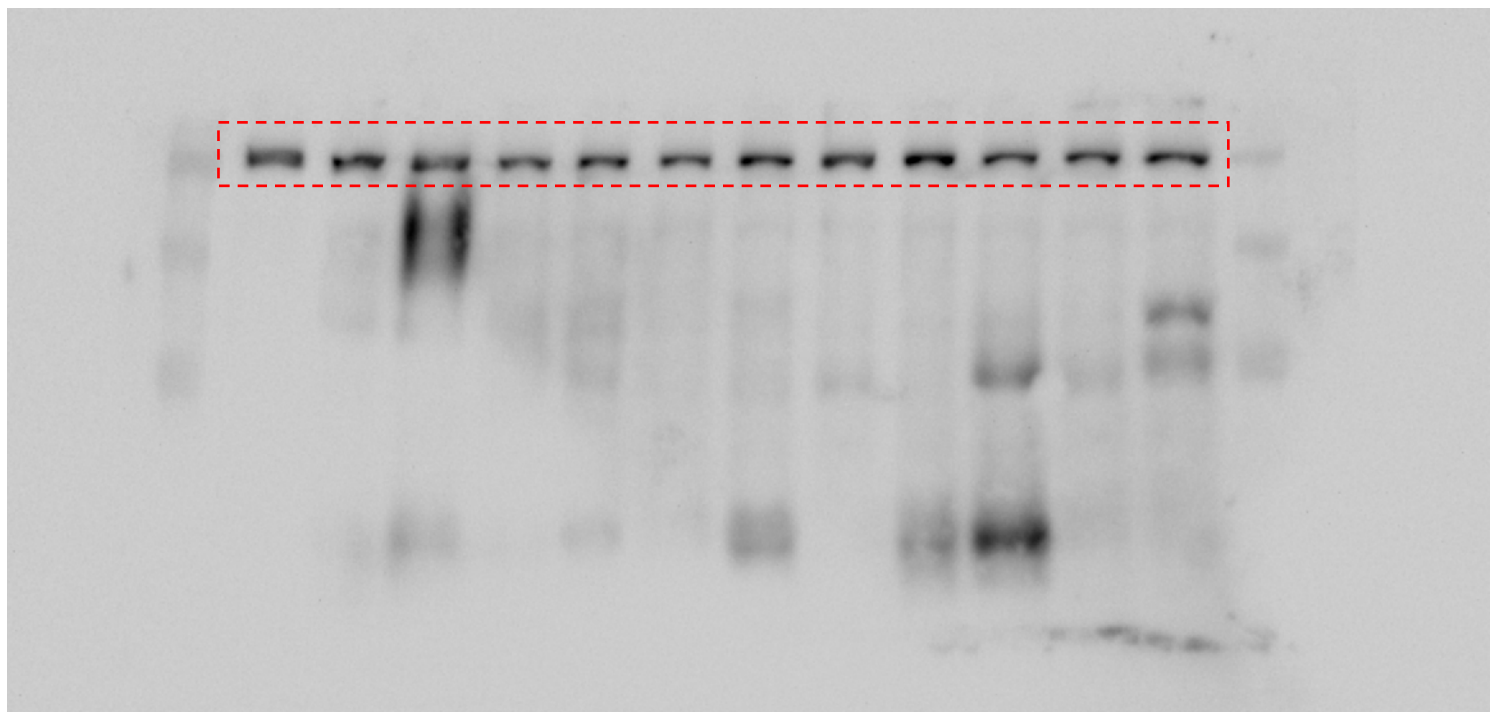

pSer935-LRRK2

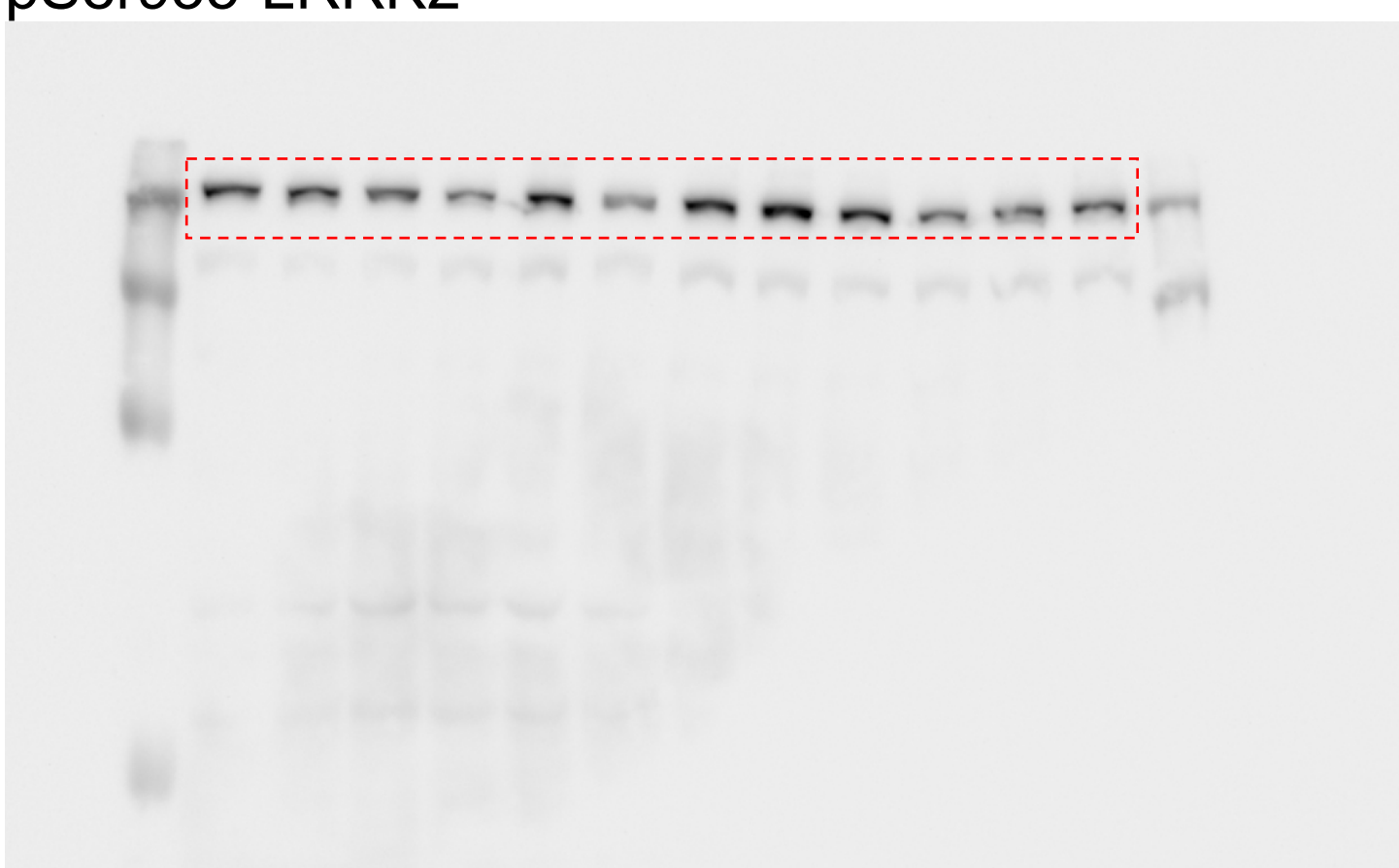

Total Rab12

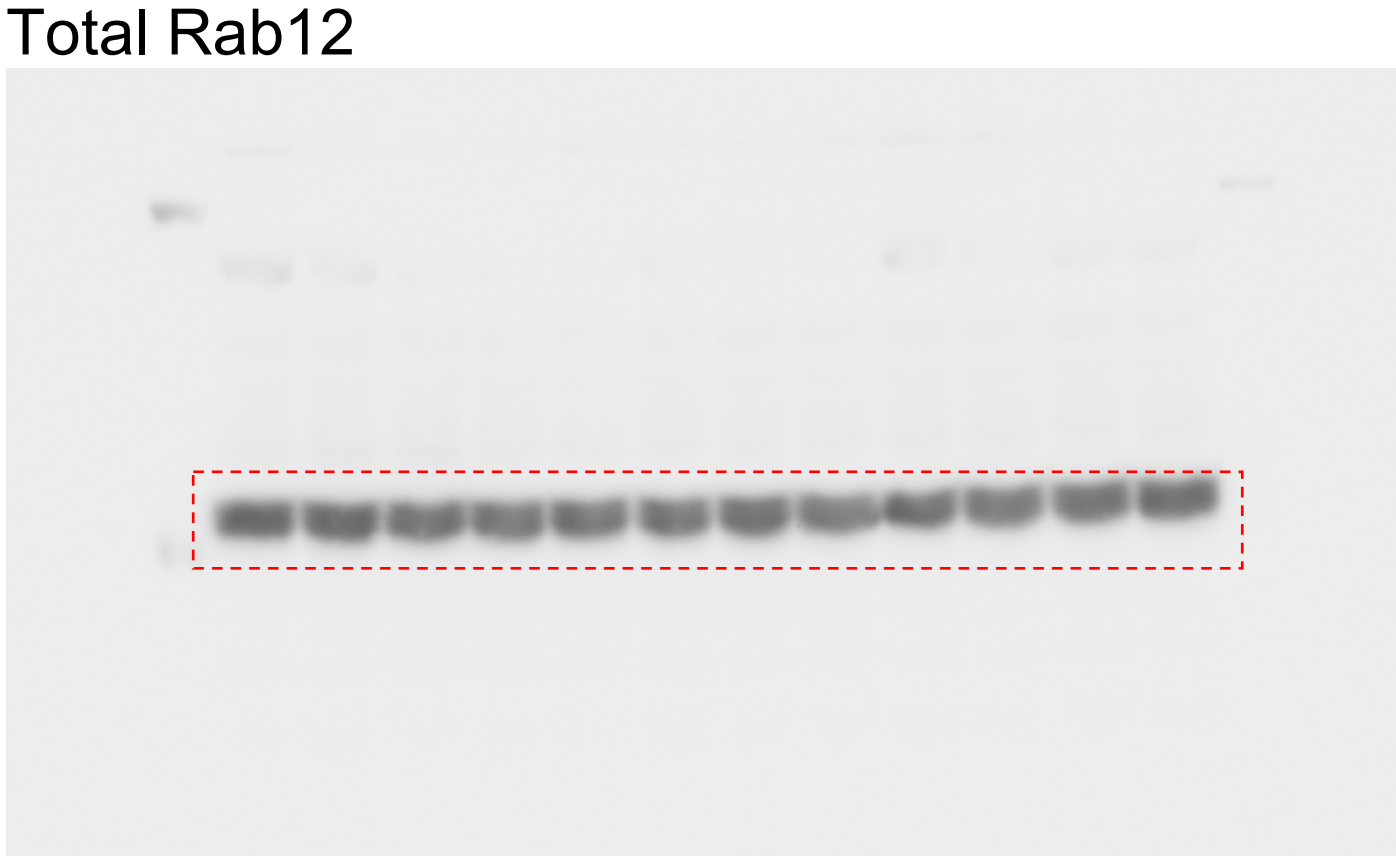

pSer106-Rab12

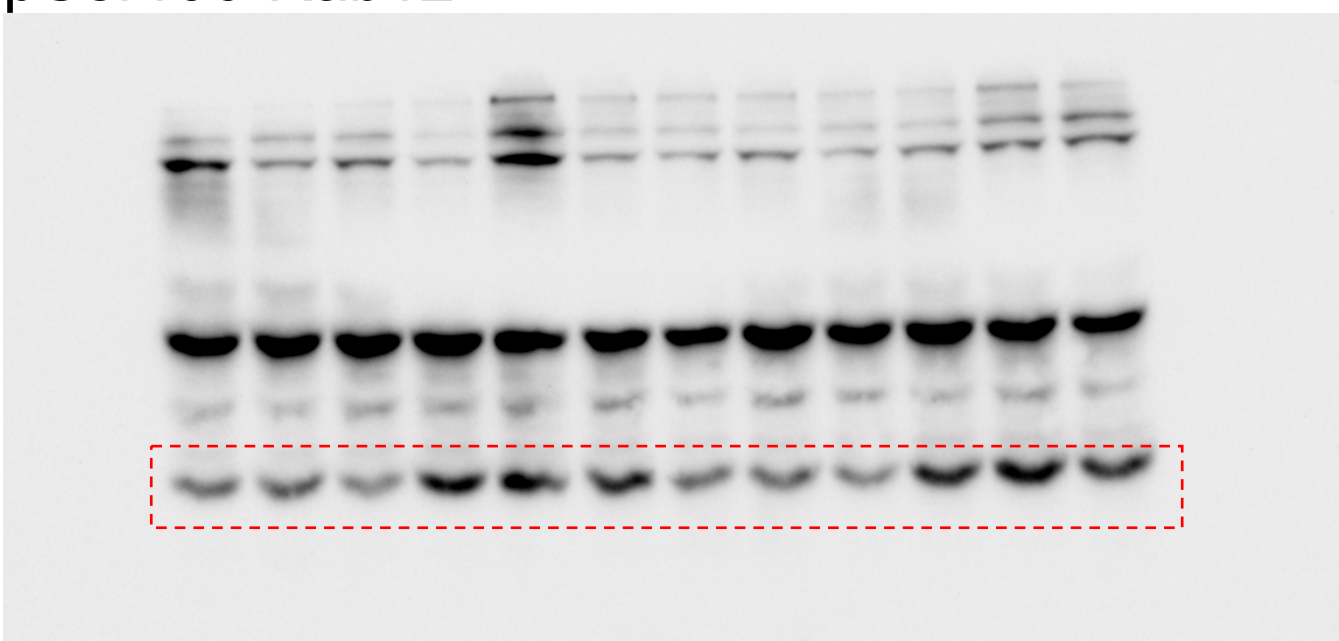

Actin

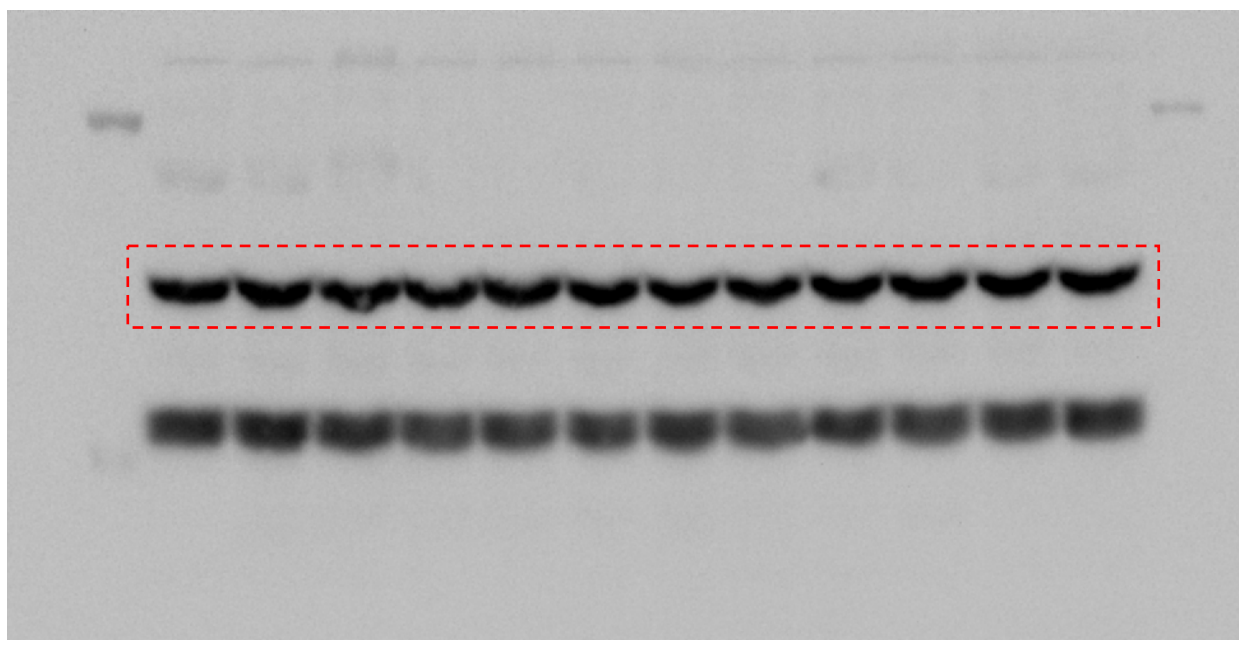

From Fig. 4h

Total LRRK2

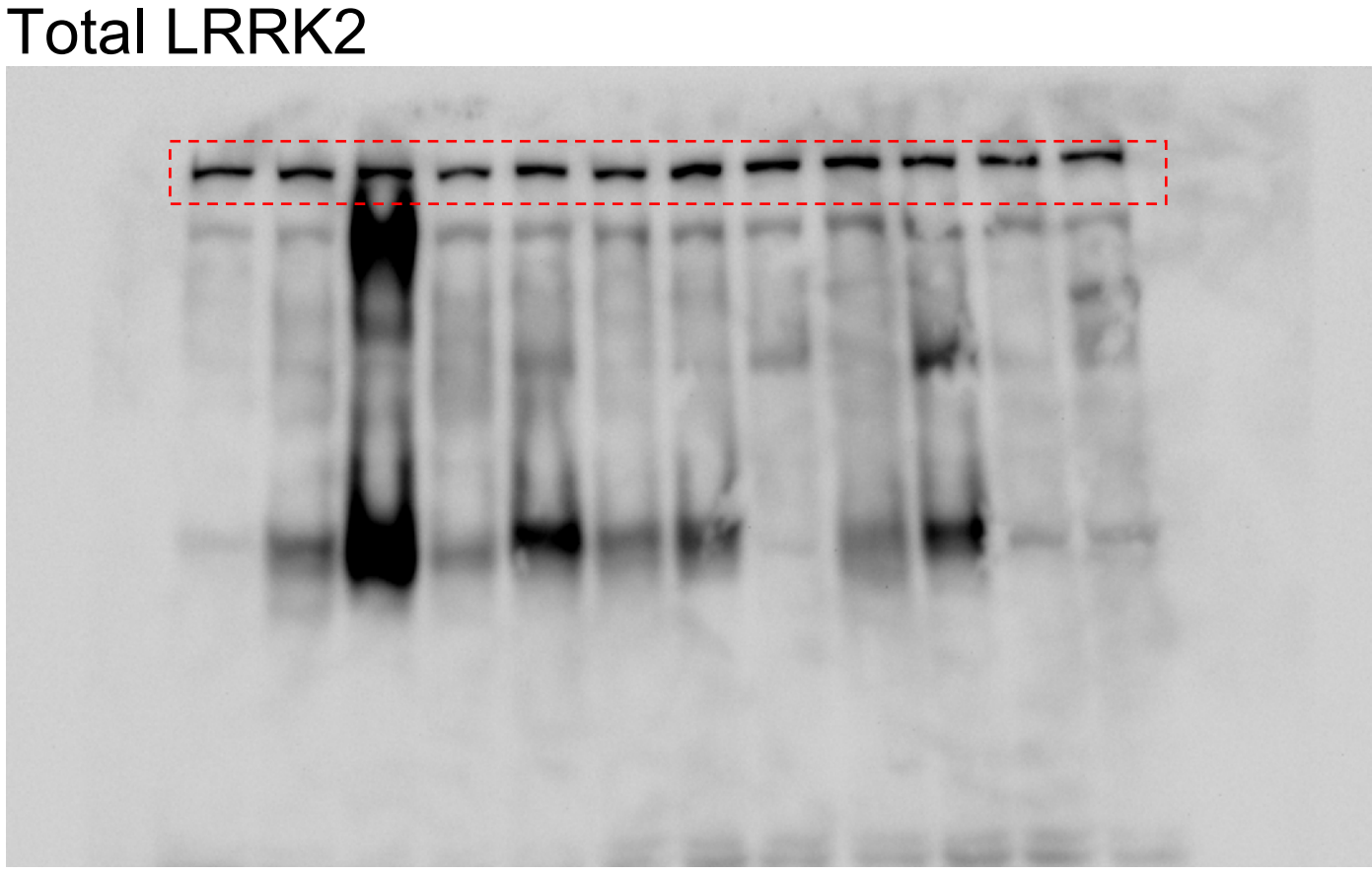

pSer935-LRRK2

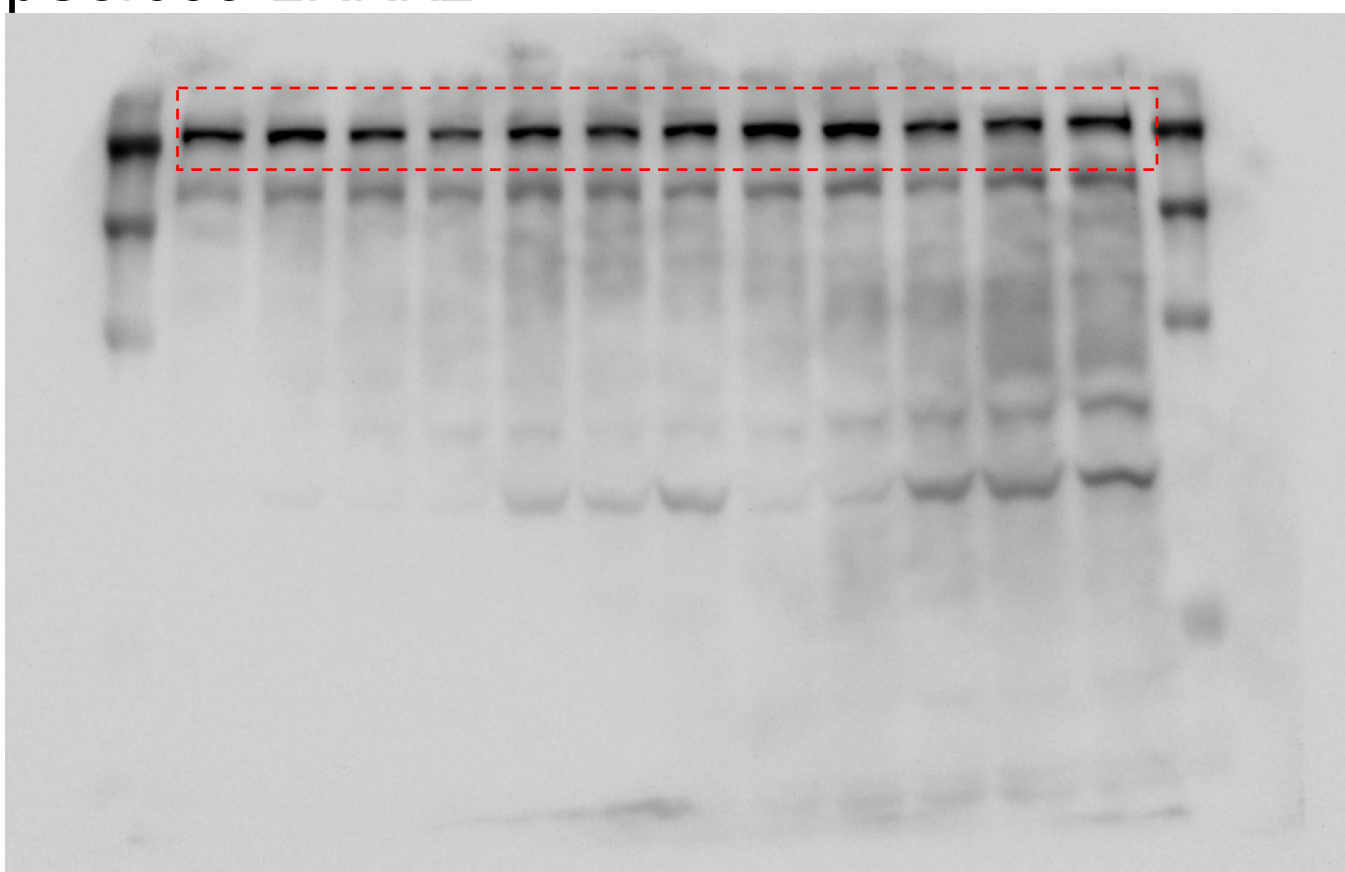

Total Rab12

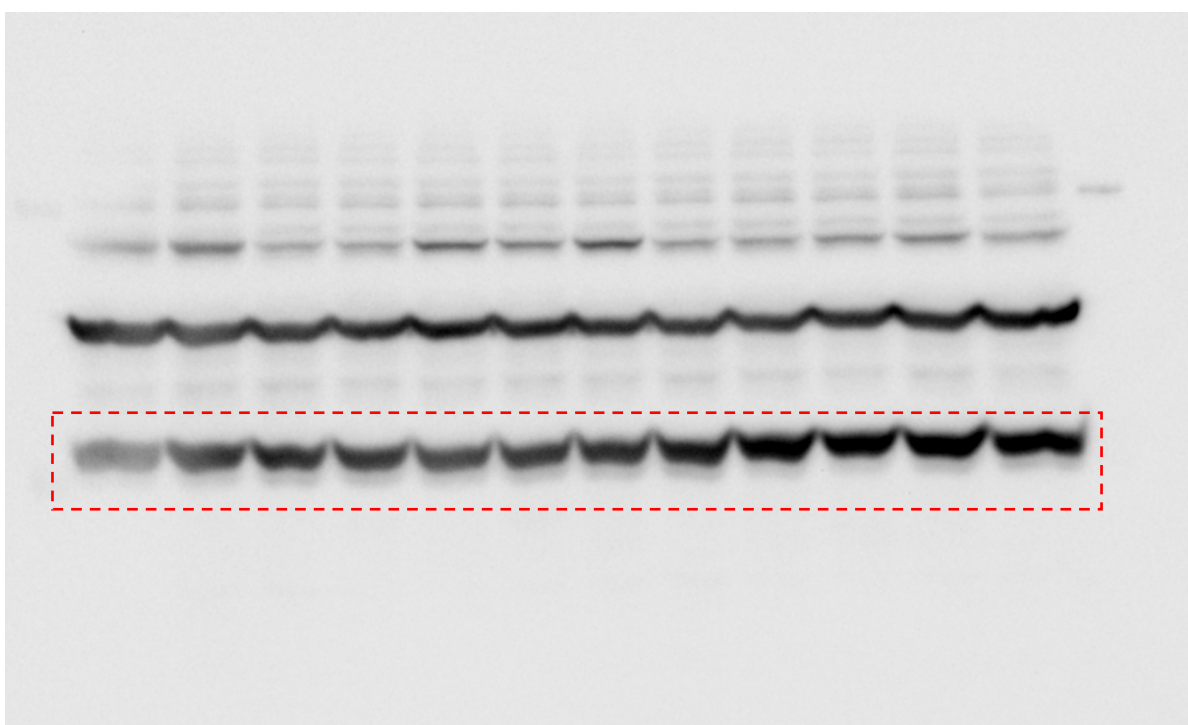

pSer106-Rab12

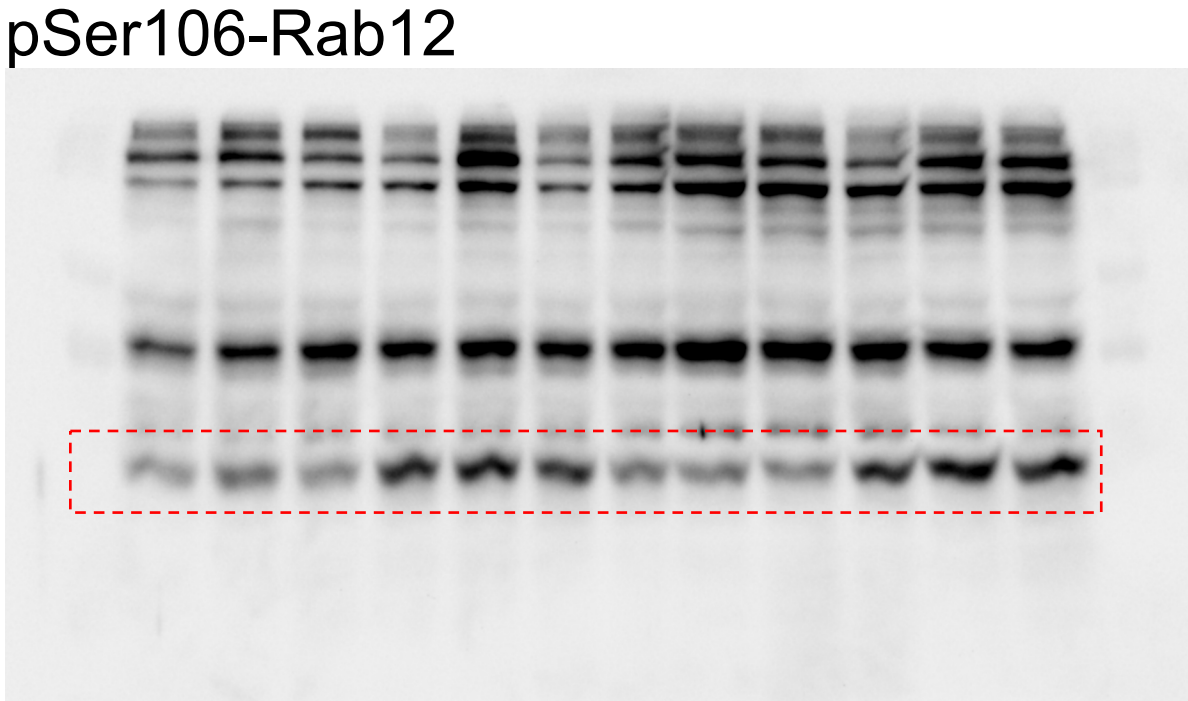

Actin

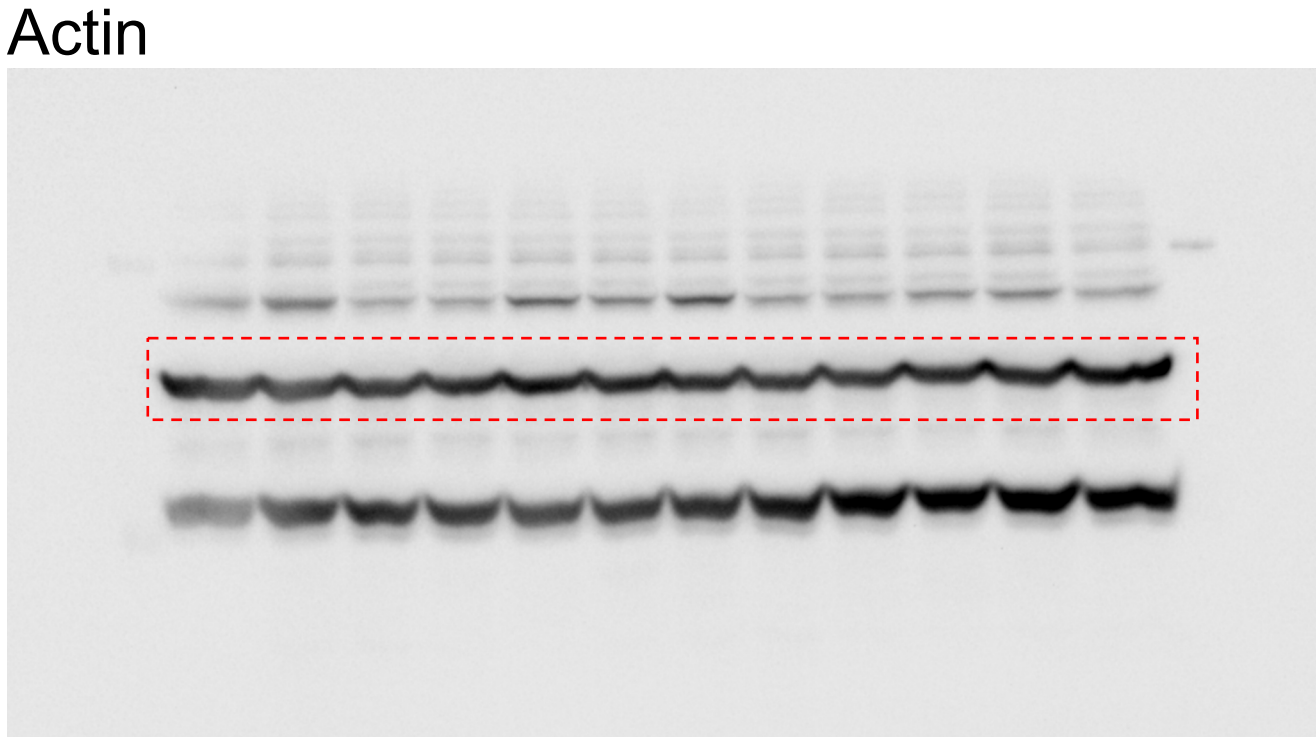

From Fig. 9b

Thymidine Phosphorylase (TP)

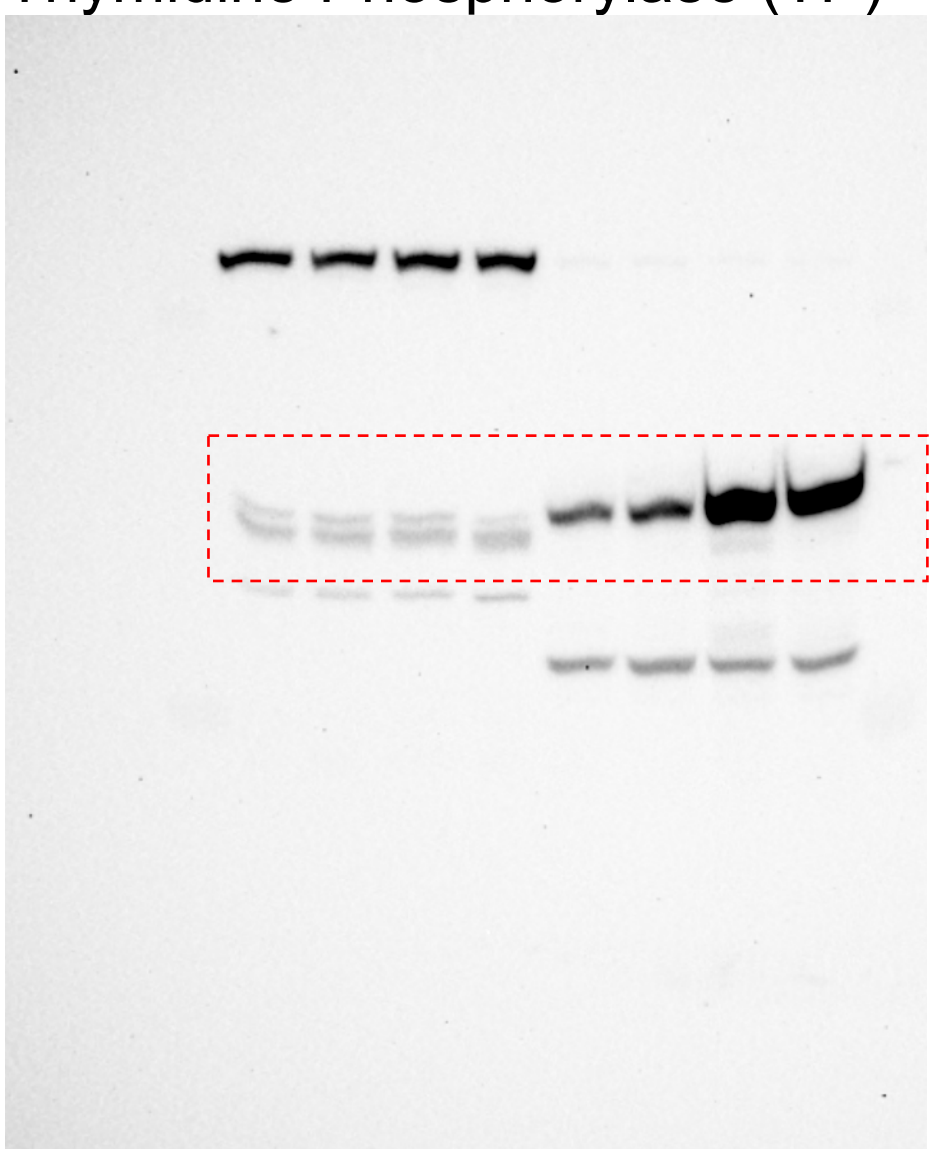

Actin

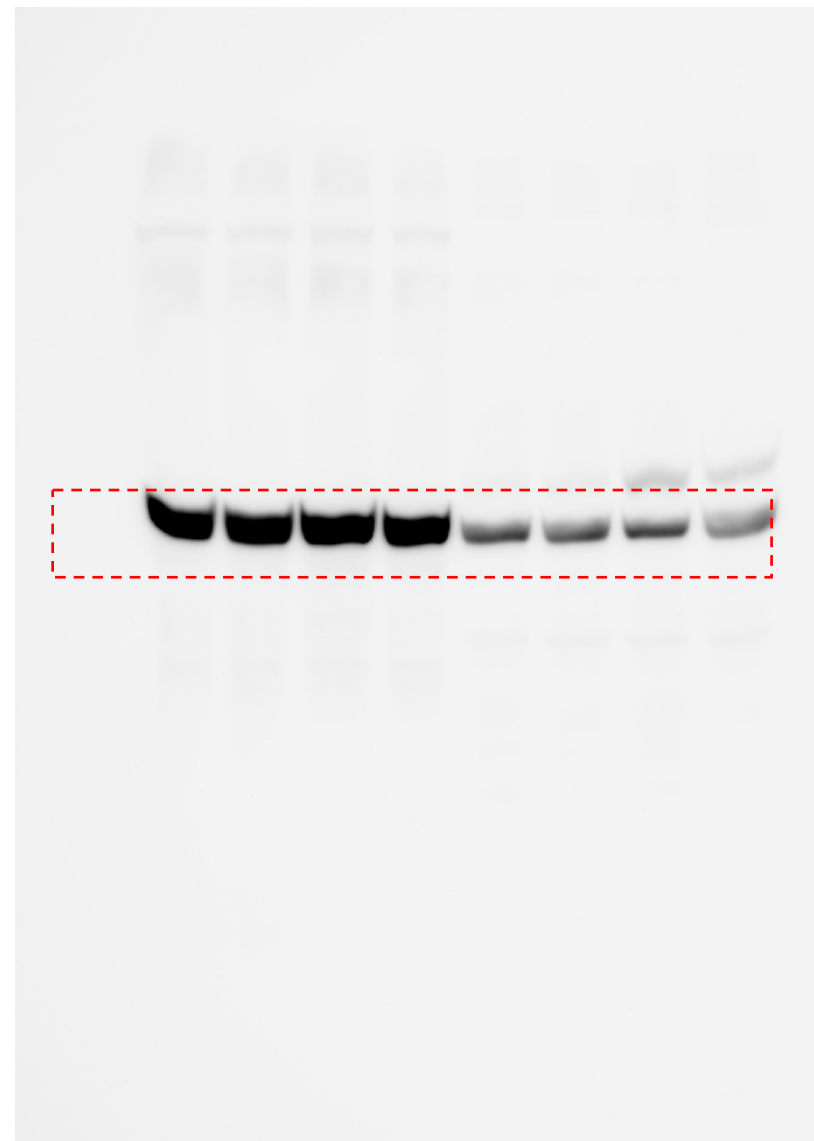

From Fig. 9f

TP (upper band) / Actin (lower band)

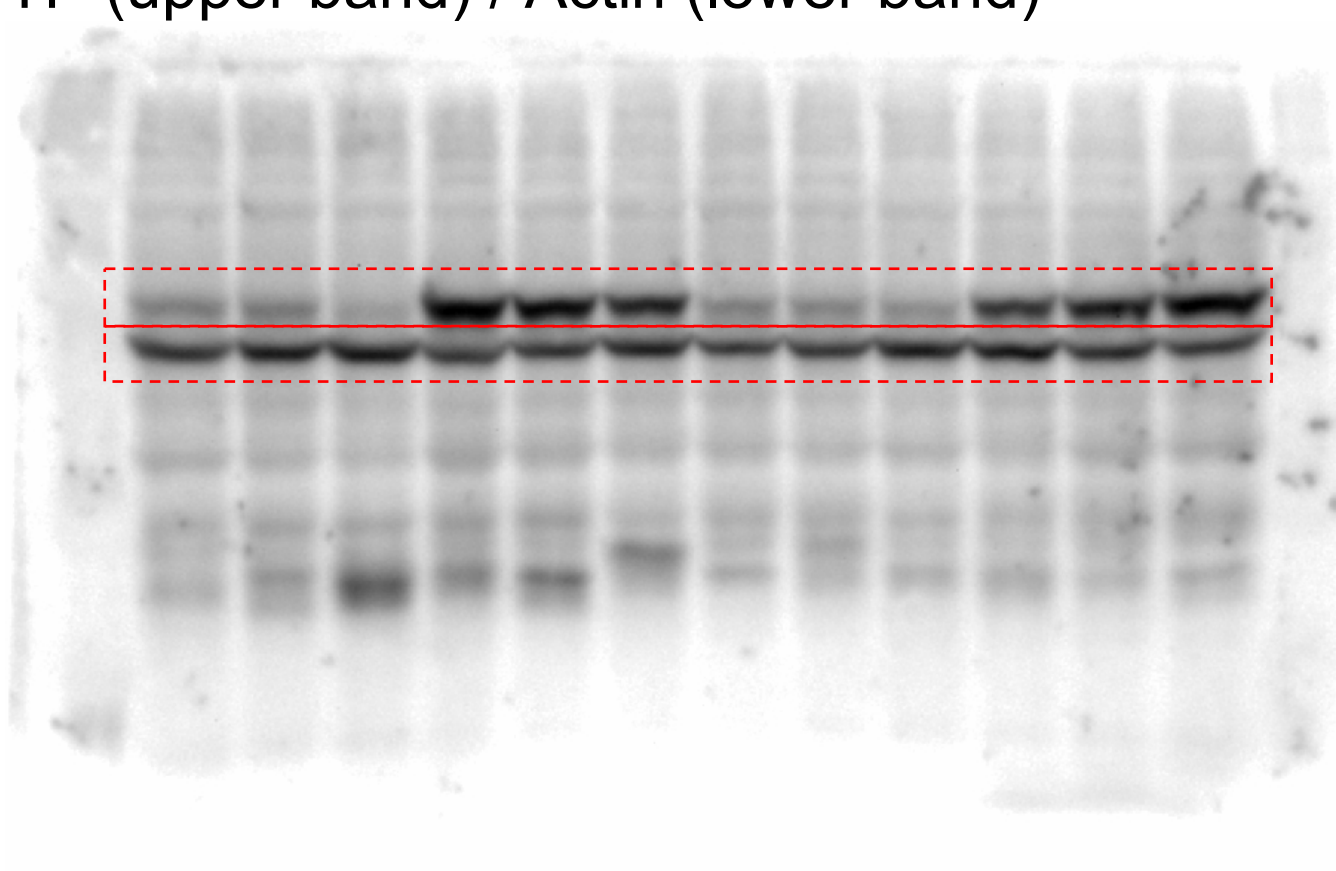

Total LRRK2

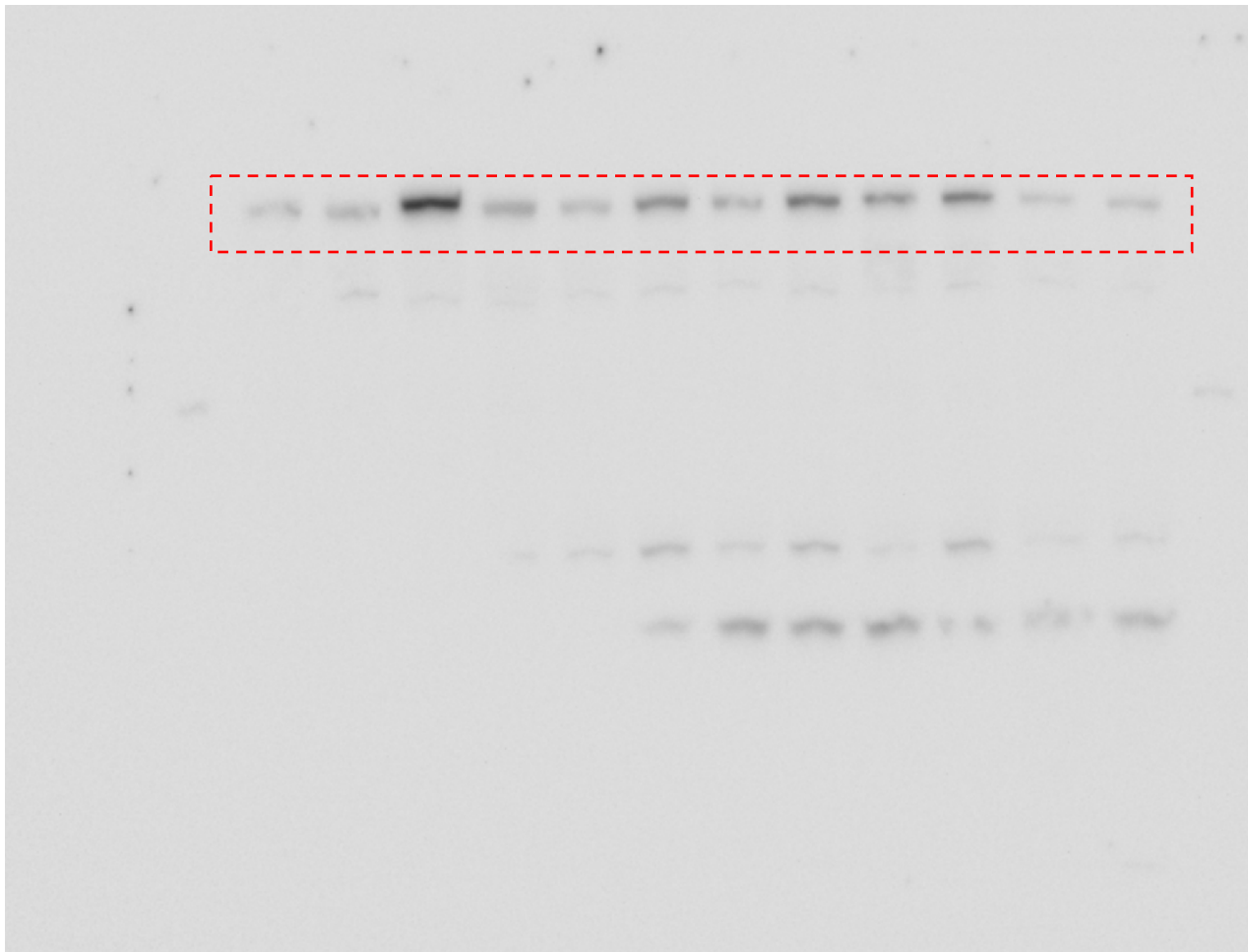

pSer1292-LRRK2

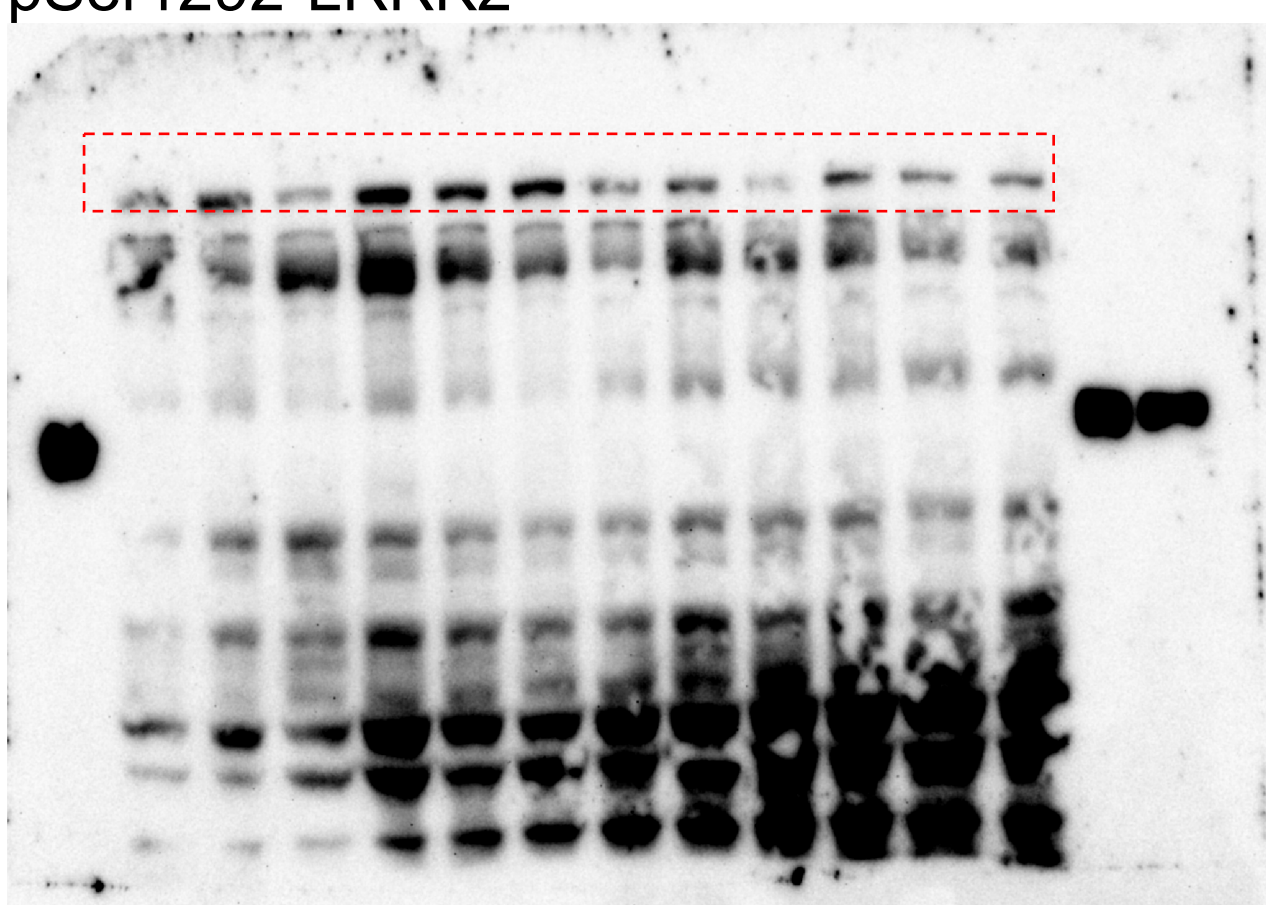

pSer935-LRRK2

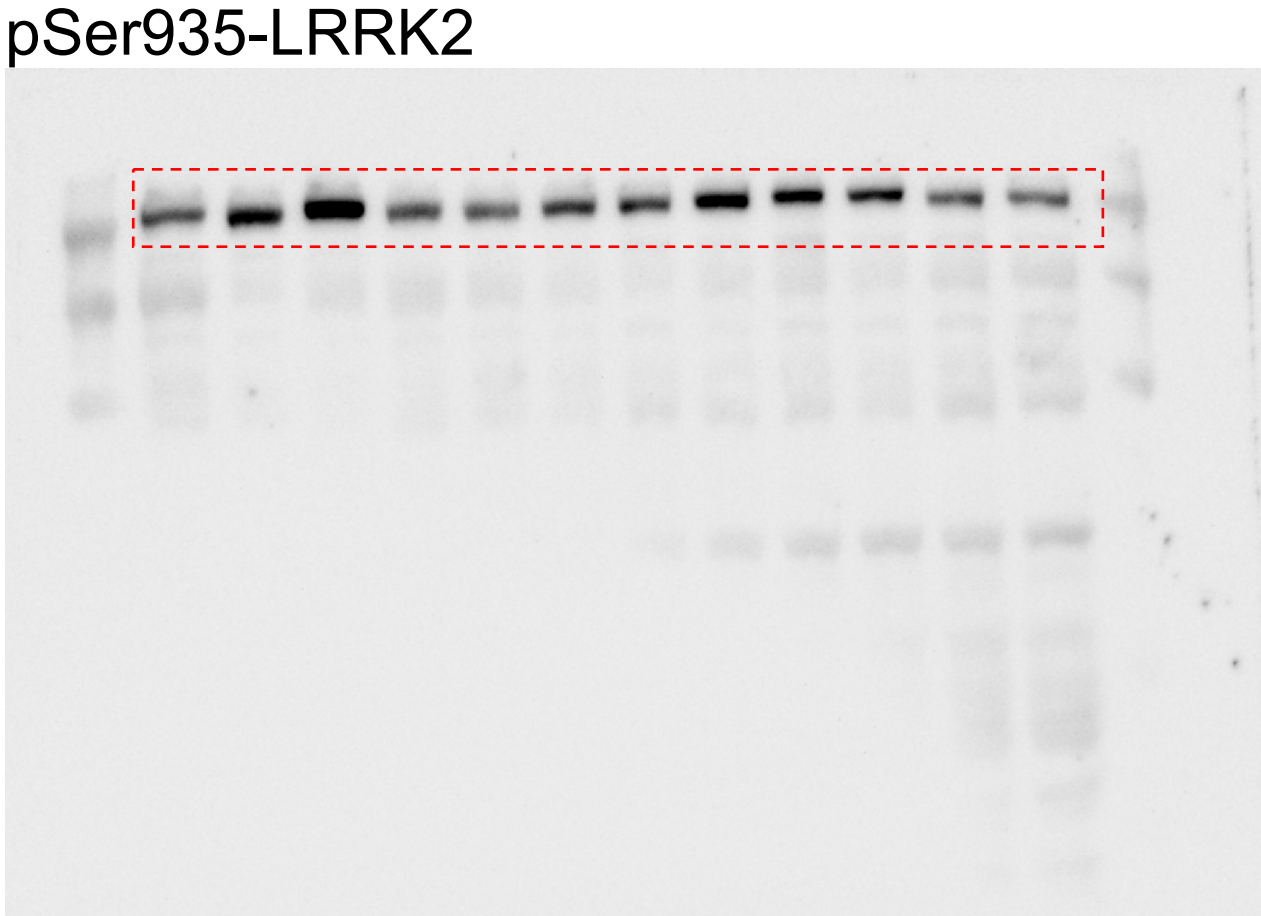

Total Rab10

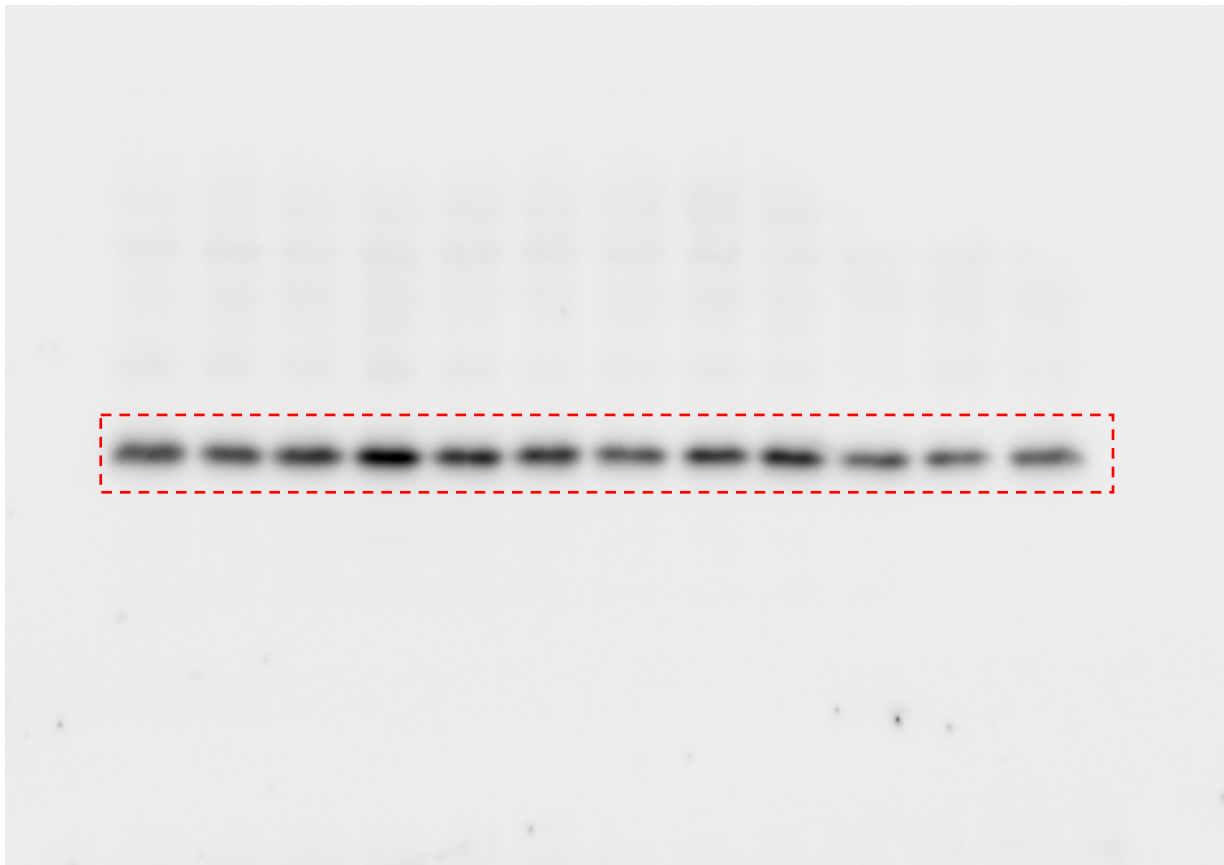

pThr73-Rab10

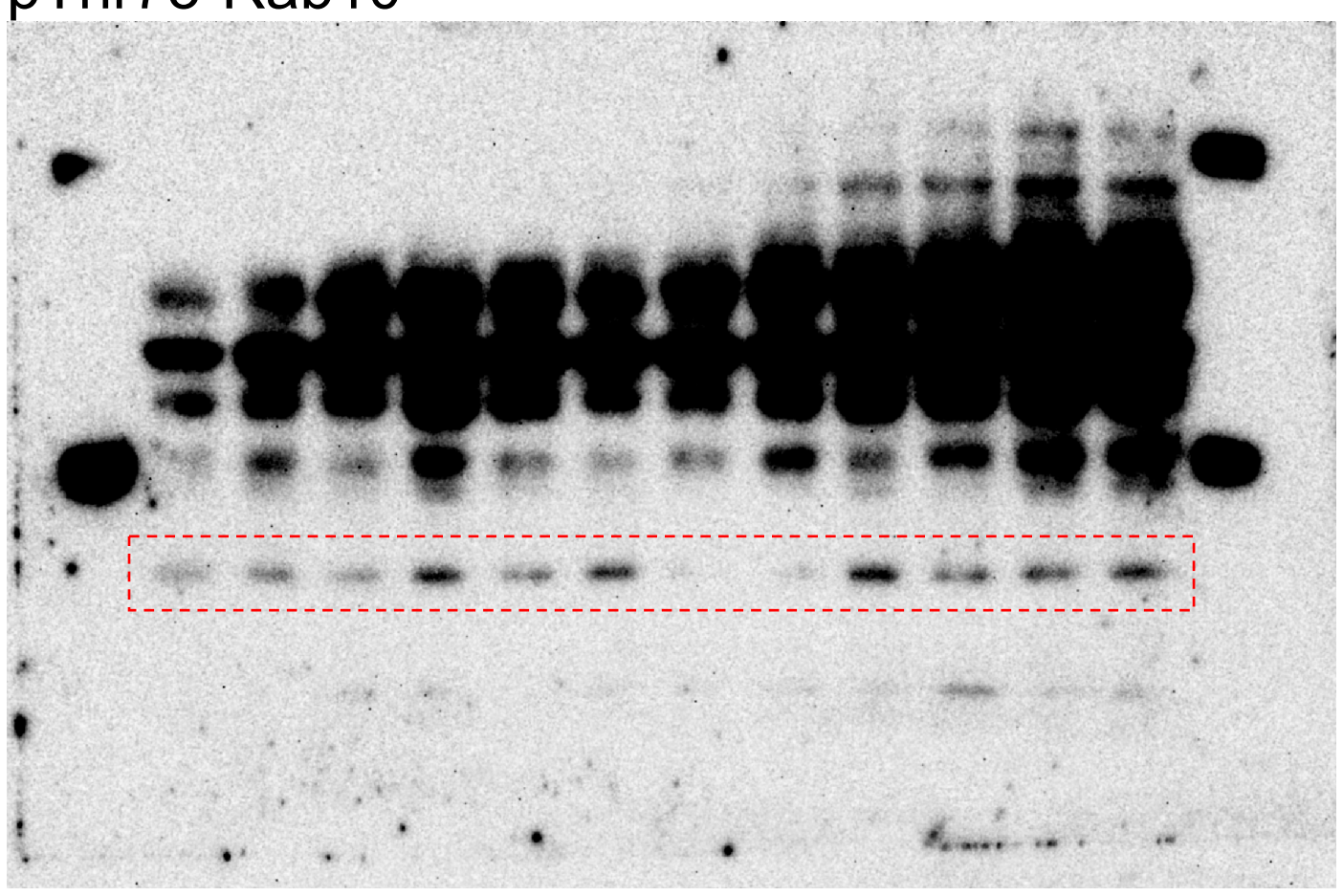

Total Rab12

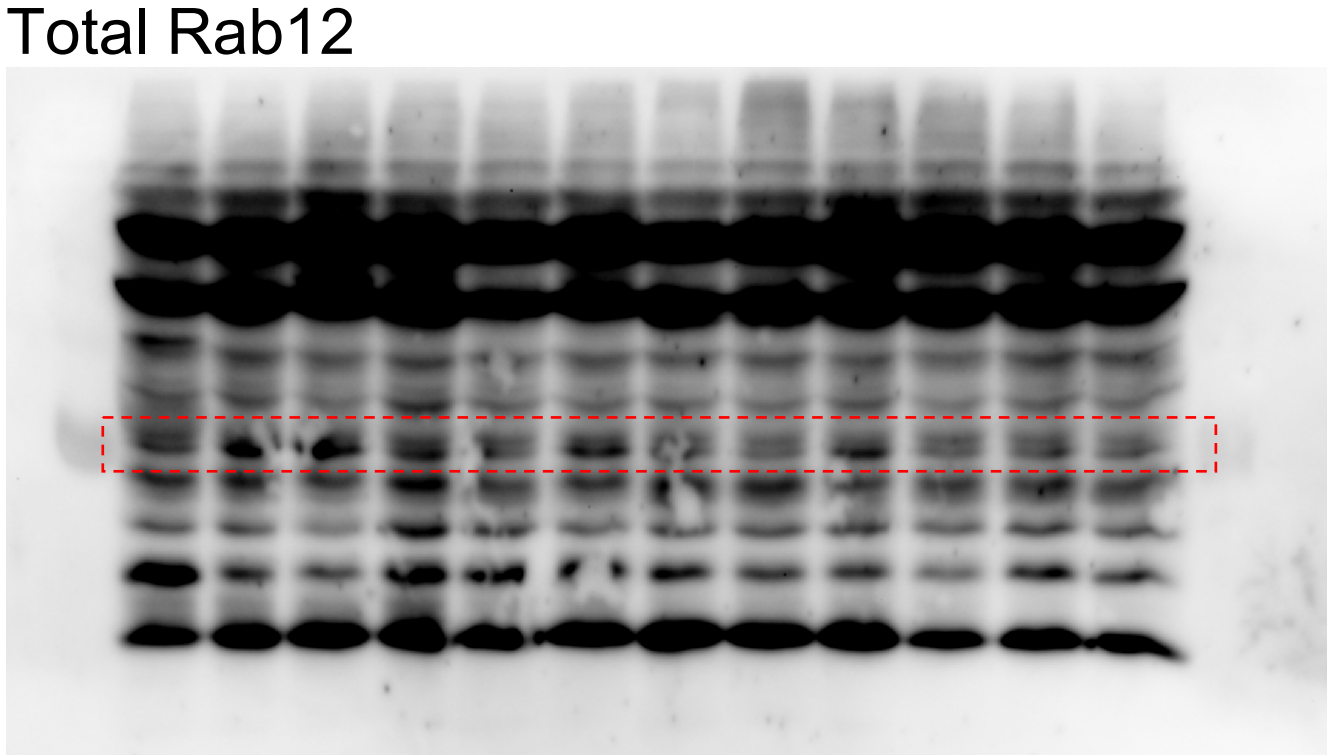

pSer106-Rab12

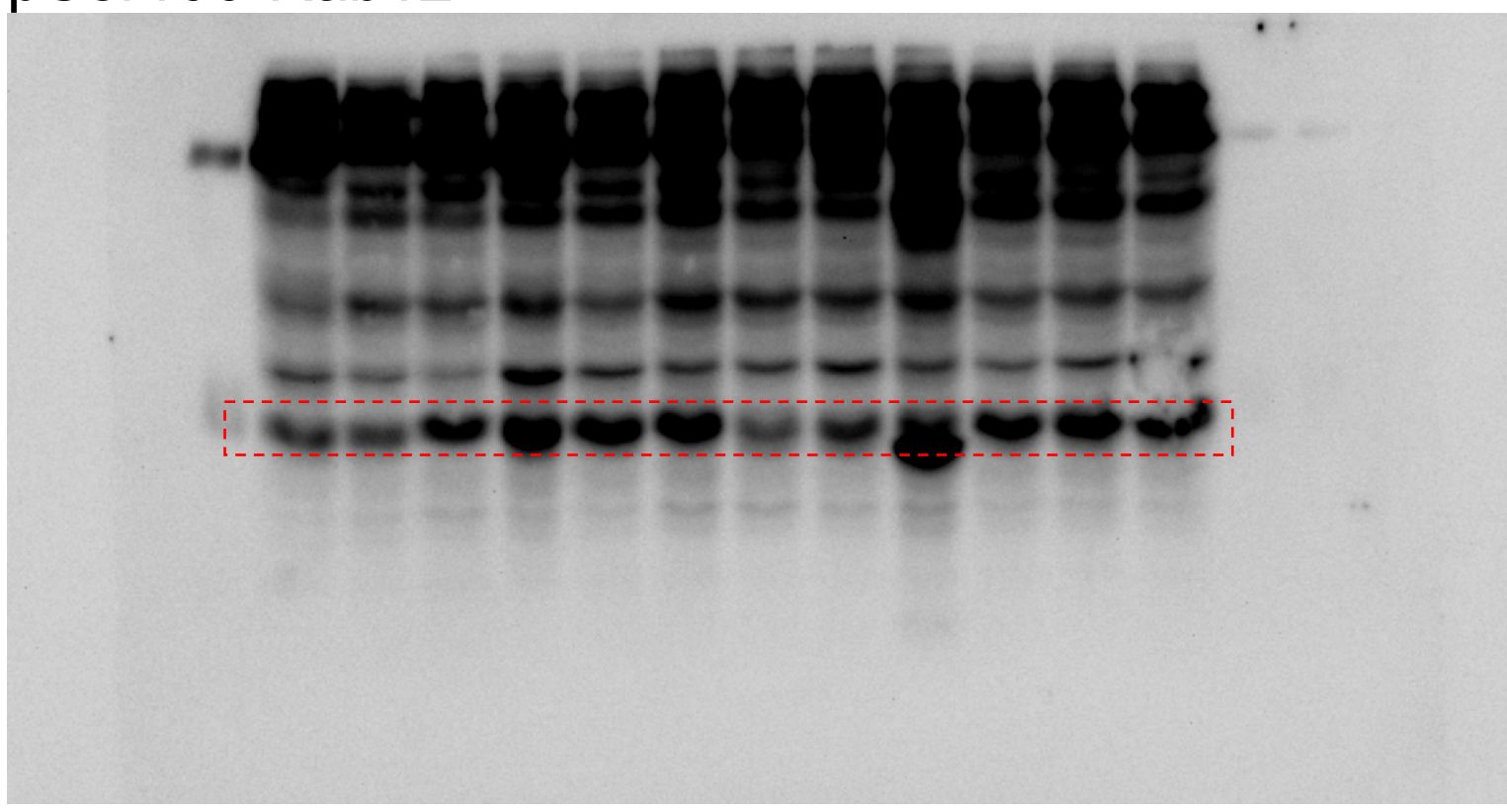

From Fig. 10x

Total LRRK2

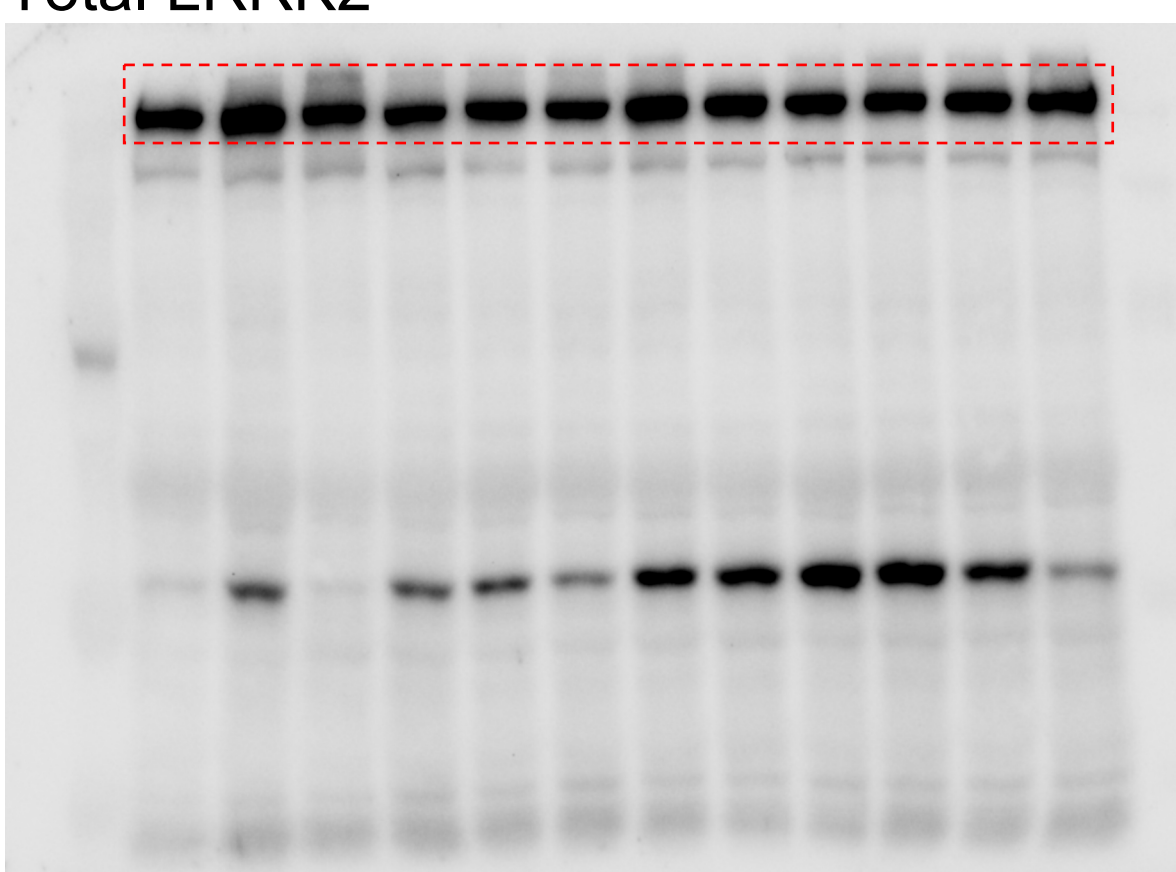

pSer935-LRRK2

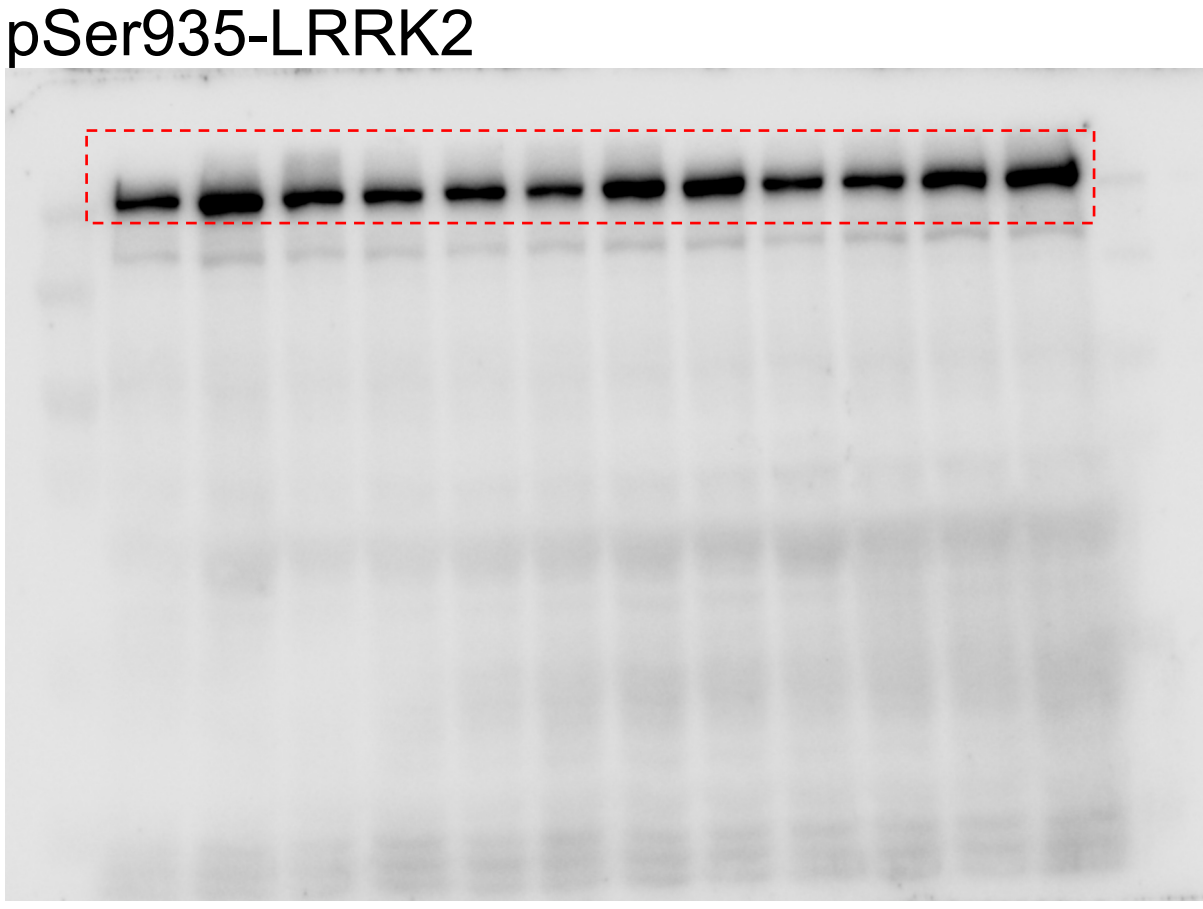

pSer1292-LRRK2

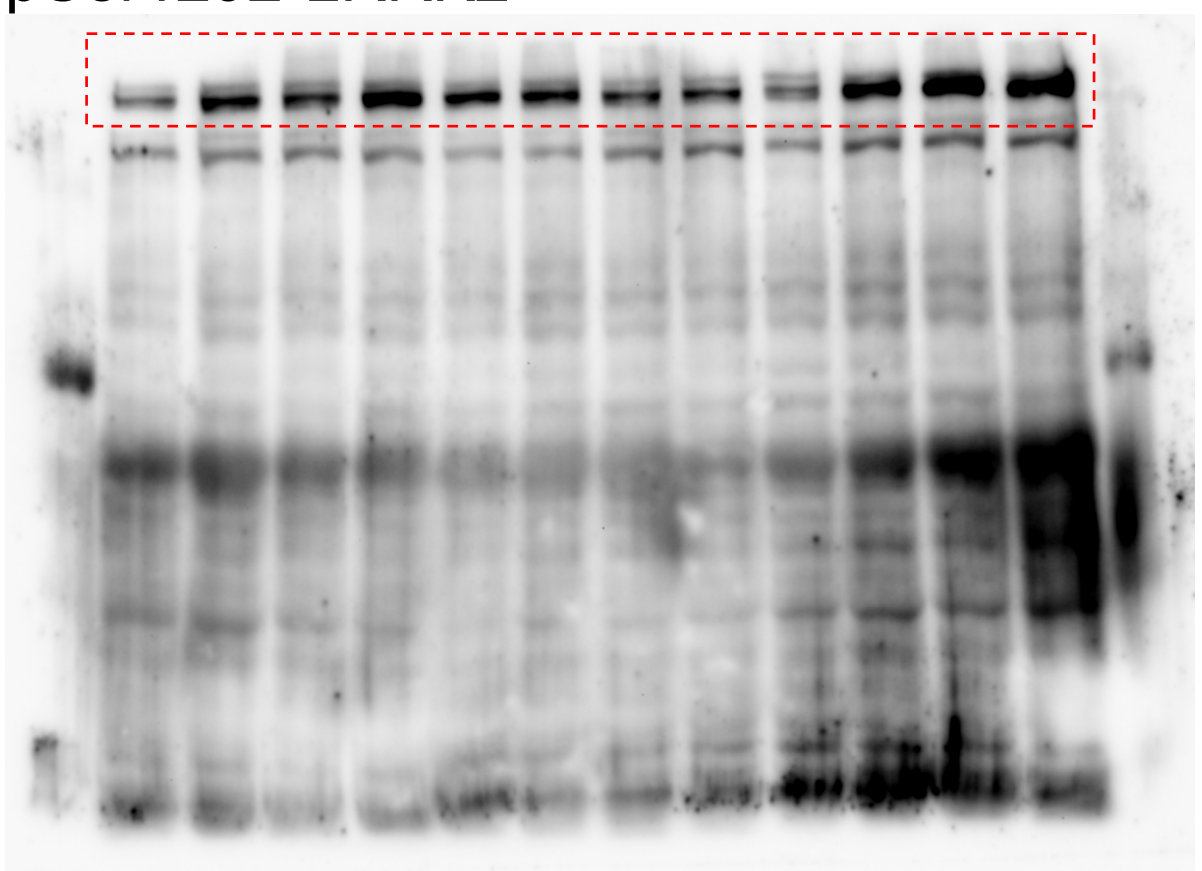

Actin

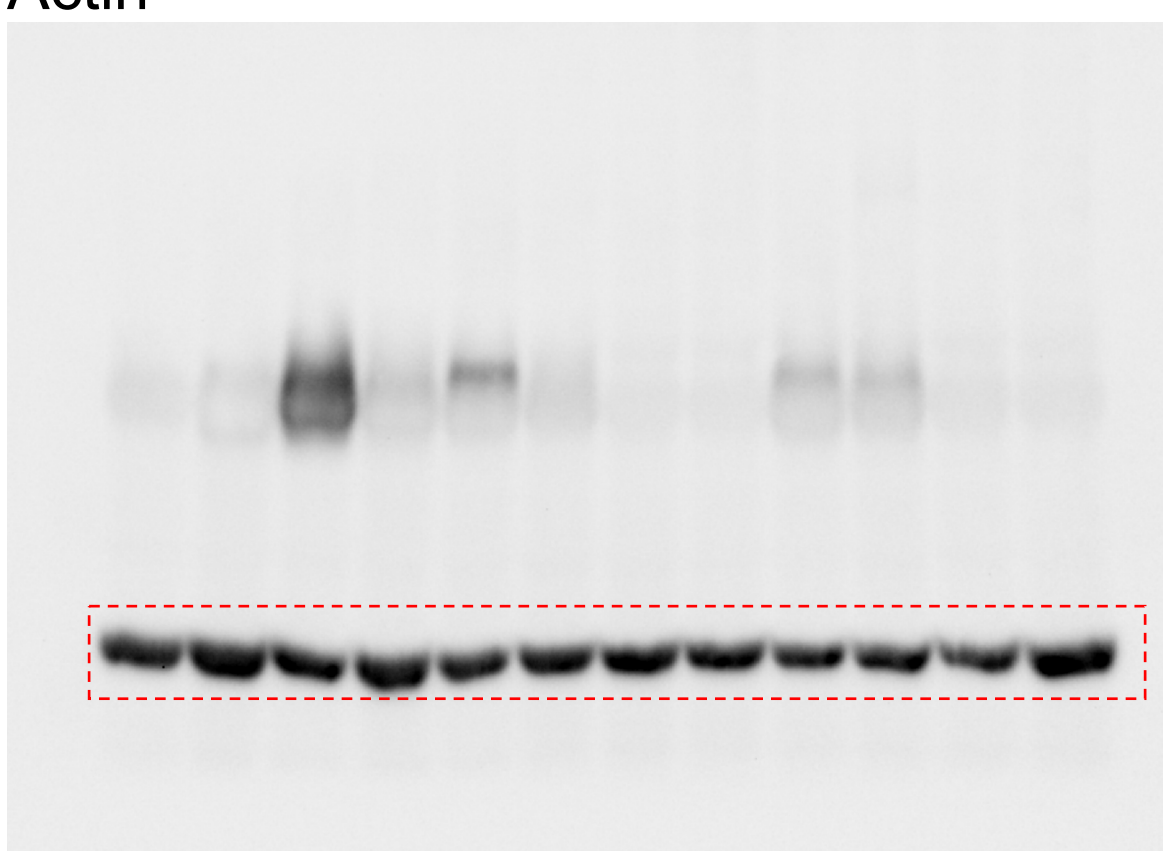

Total Rab8a

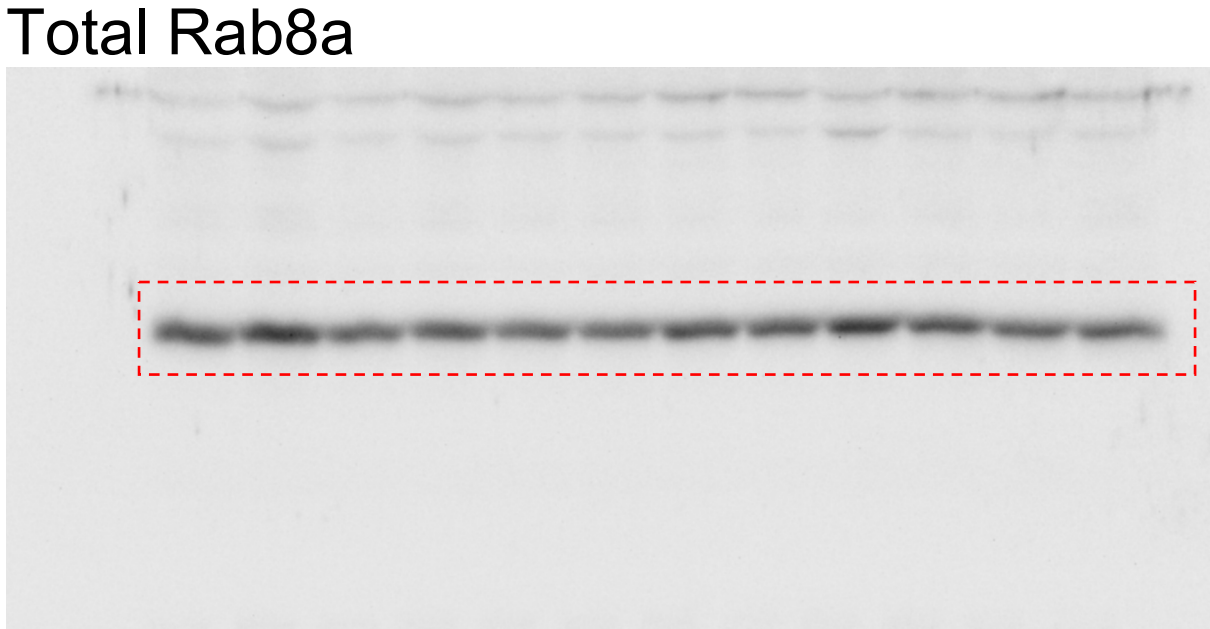

pThr72-Rab8a

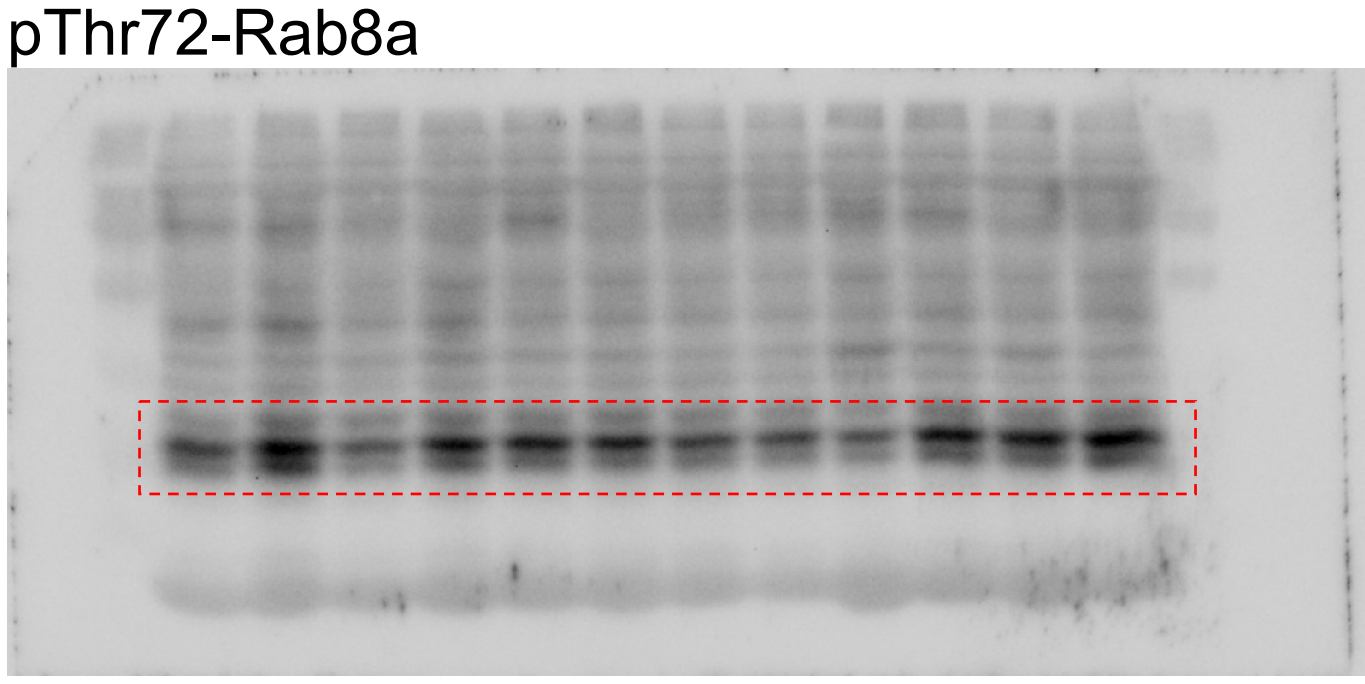

Total Rab10

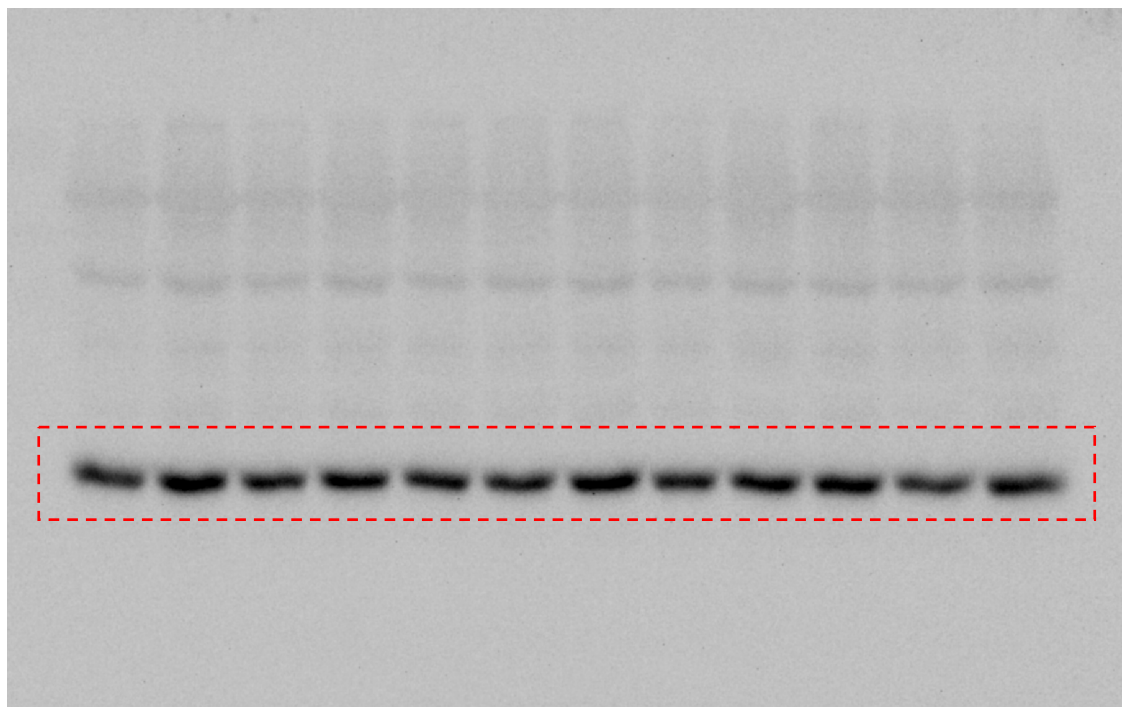

pThr73-Rab10

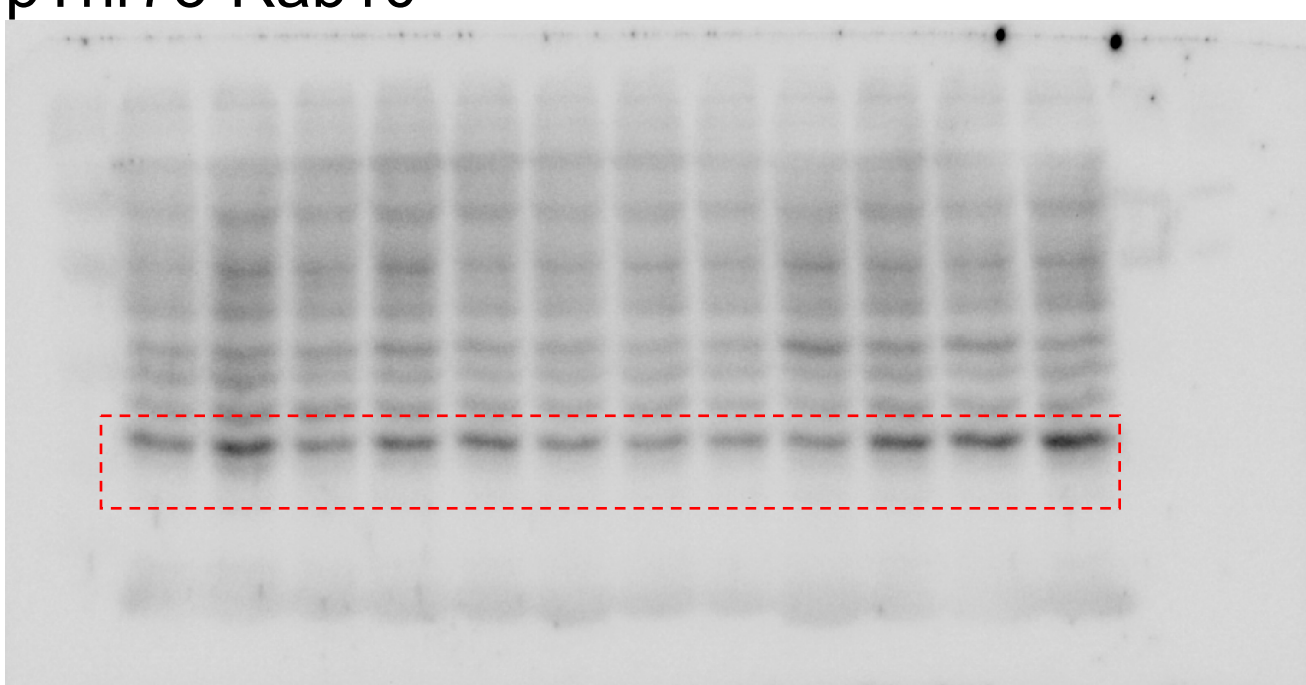

Total Rab12

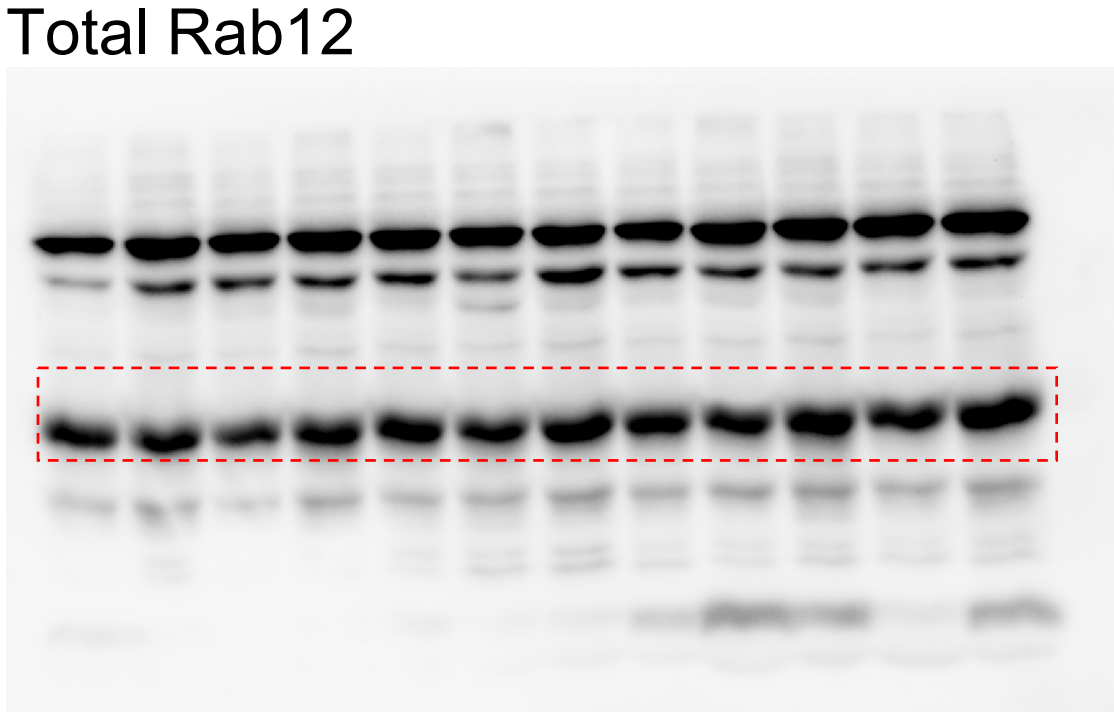

pSer106-Rab12

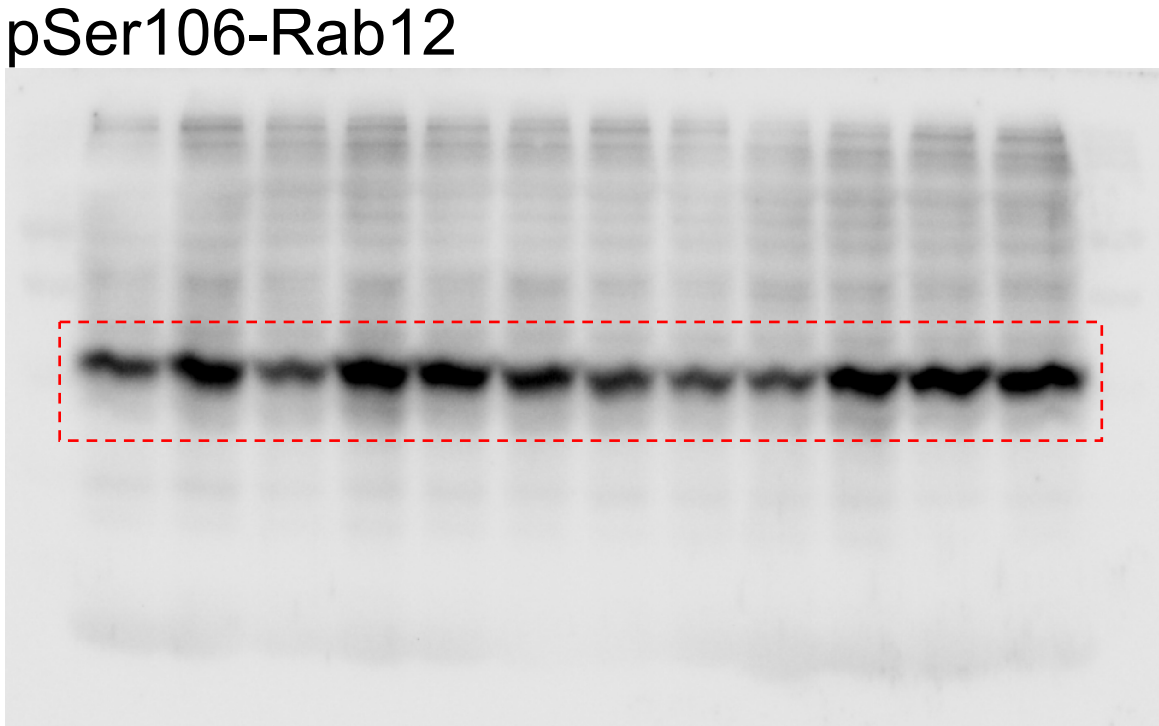

Actin

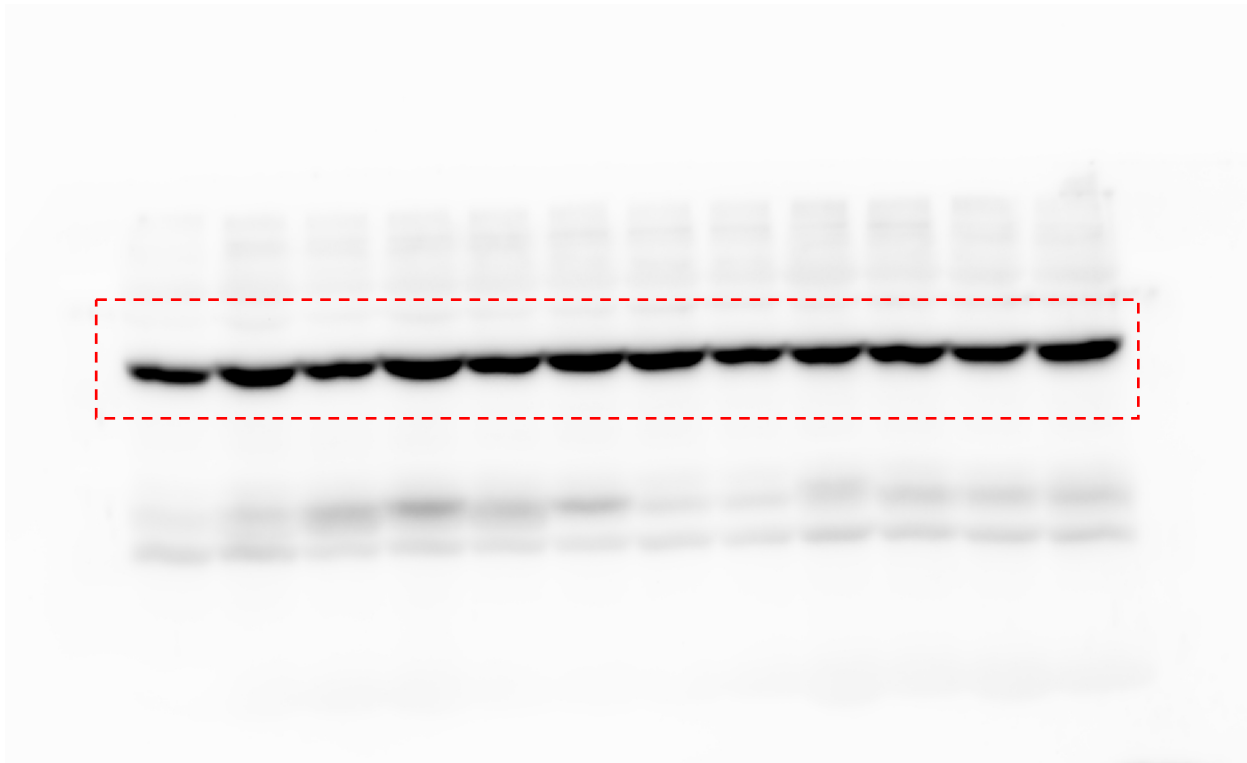

Supplement: Supplementary file 1 [file 41531_2026_1465_MOESM1_ESM.pdf]
